# Wood type influences tree responses to recurring hot and dry summer tree water deficits across European forests

**DOI:** 10.1007/s10342-026-01956-9

**Published:** 2026-10-09

**Authors:** Laura Nikinmaa, Annemiek I. Stegehuis, Marcus Lindner, Roman Zweifel, Adriaan J. Teuling, Georg von Arx, Koenraad Van Meerbeek, Roberto L. Salomón, Andreas Bär, Michal Bellan, Daniel Berveiller, Miroslav Blaženec, Vojtěch Čada, Vinicio Carraro, Sébastien Cecchini, Marco Conedera, Nicolas Delpierre, Sylvain Delzon, Ľubica Ditmarová, Johannes Edvardsson, Stefan Ehekircher, Patrick Fonti, Alicia Forner-Sales, Marta Galvagno, Andrea Ganthaler, Rainer Hentschel, Pavel Janda, Marek Ježík, Hans-Peter Kahle, Jean Kempf, Jan Krejza, Łukasz Kuberski, François Lebourgeois, Bas Lerink, Jean-Marc Limousin, Sophie Lorentz, Radim Matula, Stefan Mayr, Martin Mikoláš, Gert-Jan Nabuurs, Walter Oberhuber, Nikolaus Obojes, Bruce Osborne, Caroline Petitjean, Roman Plichta, Cyrille B. K. Rathgeber, Ute Sass-Klaassen, Matthew Saunders, Marko Smiljanic, Dominik F. Stangler, Kathy Steppe, Krzysztof Stereńczak, Marko Stojanović, Katarína Střelcová, Jan Světlík, Miroslav Svoboda, Brian Tobin, Volodymyr Trotsiuk, Josef Urban, Fernando Valladares, Monika Vejpustková, Lorenz Walthert, Martin Wilmking, Ewa Zin, Junliang Zou, Bart Muys

**Affiliations:** 1 Natural Resources Institute Finland, Latokartanonkaari 9, 00790 Helsinki, Finland; 2 Laboratoire de Geologie, IPSL, CNRS UMR 8538, École Normale Superieur, PSL University, 24 Rue Lhomond, 75231 Paris, France; 3 European Forest Institute, Platz der Vereinten Nationen 7, 53113 Bonn, Germany; 4 Swiss Federal Institute for Forest, Snow and Landscape Research WSL, Zürcherstrasse 111, 8903 Birmensdorf, Switzerland; 5 Hydrology and Environmental Hydraulics Group, Wageningen University, P.O. Box 47, 6700 AA Wageningen, The Netherlands; 6 Department of Earth and Environmental Sciences, KU Leuven, Celestijnenlaan 200E, 3001 Leuven-Heverlee, Belgium; 7 KU Leuven Plant Institute, Celestijnenlaan 200E, 3001 Leuven-Heverlee, Belgium; 8 Departamento de Sistemas y Recursos Naturales, Universidad Politécnica de Madrid, E.T.S.I. de Montes, Forestal y del Medio Natural, Universidad Politécnica de Madrid, Ciudad Universitaria s/n., 28040 Madrid, Spain; 9 Department of Botany, University of Innsbruck, Sternwartestraße 15, 6020 Innsbruck, Austria; 10 Department of Forest Ecology, Mendel University in Brno, Zemědělská 3, 61300 Brno, Czech Republic; 11 Université Paris-Saclay, CNRS, AgroParisTech, Ecologie Société et Evolution, Bât. 680 - 12, route 128, 91190 Gif- sur-Yvette, France; 12 Institute of Forest Ecology, Slovak Academy of Sciences, Ľ. Štúra 2, 96001 Zvolen, Slovakia; 13 Czech University of Life Sciences, Kamycka 129, 165 21 Prague 6 - Suchdol, Czech Republic; 14 Department of Land, Environment, Agriculture and Forestry, University of Padua, Viale dell’Università 16, 35020 Legnaro, Italy; 15 Office National des Forêts, Département Recherche Développement et Innovation, Boulevard de Constance, 77300 Fontainebleau, France; 16 Réseau National de suivi à long terme des ECOsystèmes FORestiers, Office National des Forêts, Boulevard de Constance, 77300 Fontainebleau, France; 17 Insubric Ecosystems Research Group, Swiss Federal Research Institute for Forest, Snow and Landscape Research WSL, Campus di Ricercaa Ramél 18, 6593 Cadenazzo, Switzerland; 18 INRAE, UMR BIOGECO, Université de Bordeaux - Bâtiment B2, Allée Geoffroy St-Hilaire, 33615 Pessac, France; 19 University of Bordeaux, UMR BIOGECO, Université de Bordeaux - Bâtiment B2, Allée Geoffroy St-Hilaire, 33615 Pessac, France; 20 Department of Earth and Environmental Sciences, Lund University, Sölvegatan 12, 223 62 Lund, Sweden; 21 University of Applied Forest Sciences Rottenburg, Schadenweilerhof, 72108 Rottenburg, Germany; 22 Faculty of Education, Universidad Internacional de La Rioja (UNIR), Avenida de la Paz 137, 26006 Logroño, La Rioja Spain; 23 Department of Biogeography and Global Change, International Global Change Laboratori (LINCGlobal), Museo Nacional de Ciencias Naturales, Consejo Superior de Investigaciones Científicas, Serrano 115 dpdo, 28006 Madrid, Spain; 24 Environmental Protection Agency of Aosta Valley (ARPA VdA), La Maladière, 48, 11020 Saint-Christophe, Aosta, Italy; 25 Eberswalde Forestry State Center of Excellence, Alfred-Möller-Str. 1, 16225 Eberswalde, Germany; 26 Chair of Forest Growth and Dendroecology, Institute of Forest Sciences, University of Freiburg, Tennenbacher Str. 4, 79106 Freiburg, Germany; 27 CEFE, University of Montpellier, CNRS, EPHE, IRD, 1919 Route de Mende, 34293 Montpellier 5, France; 28 Global Change Research Institute of the Czech Academy of Sciences, Bělidla 986/4a, 603 00 Brno, Czech Republic; 29 Department of Natural Forests, Forest Research Institute (IBL), 6 Park Dyrekcyjny St, 17-230, Białowieża, Poland; 30 Université de Lorraine, AgroParisTech, INRAE, SILVA, 54000 Nancy, France; 31 Wageningen Environmental Research, Wageningen University and Research, P.O. Box 47, 6700 AA Wageningen, The Netherlands; 32 Eurac Research, Institute for Alpine Environment, Viale Druso 1, 39100 Bolzano, Italy; 33 UCD School of Agriculture and Food Science, Agriculture and Food Science Centre, University College Dublin, Belfield, Dublin 4, Ireland; 34 UCD Earth Institute, University College Dublin, Belfield, Dublin 4, Ireland; 35 Department of Forest Botany, Dendrology and Geobiocoenology, Mendel University in Brno, Zemědělská 1, 613 00 Brno, Czech Republic; 36 Department of Experimental Biology, Faculty of Science, Masaryk University, Kotlářská 267/2, 611 37 Brno, Czech Republic; 37 Forest and Nature Management, Van Hall Larenstein University of Applied Sciences, P.O. Box 9001, 6880 GB Velp, The Netherlands; 38 Forest Ecology and Forest Management Group, Wageningen University and Research, P.O. Box 47, 6700 AA Wageningen, The Netherlands; 39 School of Natural Sciences, Botany Discipline, Trinity College Dublin, College Green, D2, Dublin, Ireland; 40 DendroGreif, Institute for Botany and Landscape Ecology, University of Greifswald, Domstraße 11, 17489 Greifswald, Germany; 41 Laboratory of Plant Ecology, Faculty of Bioscience Engineering, Ghent University, Coupure links 653, 9000 Ghent, Belgium; 42 Department of Geomatics, Forest Research Institute, Sekocin Stary, 3 Braci Leśnej St, 05-090 Raszyn, Poland; 43 Technical University in Zvolen, Masarykova 24, 96001 Zvolen, Slovakia; 44 Museo Nacional de Ciencias Naturales, Consejo Superior de Investigaciones Científicas, Serrano 115 dpdo, 28006 Madrid, Spain; 45 Universidad Rey Juan Carlos (URJC), Calle Tulipán s/n., 28933 Mostoles, Madrid, Spain; 46 Forestry and Game Management Research Institute, Strnady 136, 252 02 Jiloviste, Czech Republic; 47 Department of Natural Forests, Dendrolab IBL, Forest Research Institute (IBL), 6 Park Dyrekcyjny St, 17-230, Białowieża, Poland; 48 Southern Swedish Forest Research Centre, Swedish University of Agricultural Sciences (SLU), Lomma, 234 22 Sweden; 49 Forestry Development Department and Climate Centre, Teagasc, Johnstown Castle, Co., Wexford, Y35 TC97 Ireland; 50 Oeschger Centre for Climate Change Research, University of Bern, Hochschulstrasse 4, 3012 Bern, Switzerland

**Keywords:** Tree water deficit, Wood anatomy, Dendrometer, European forests, Tree stress, Drought recurrence

## Abstract

Increasingly frequent summer droughts are intensifying stem dehydration across European forests, constraining tree growth and resilience. Even mild water-related stress can inhibit tree growth, although its impact is species-specific. Tree water deficit (TWD), a measure deduced from radial stem contraction during drought, reflects stem water status and can be used as a proxy for the physiological drought stress experienced by trees. Using high-resolution dendrometer measurements representing 29 tree species from 130 forest sites across Europe from 2003 to 2018, we examined how the length of TWD periods during summer is related to their recurrence within the same year, to TWD intensity, to climate factors, and to wood type (i.e., tracheid-based coniferous, ring-porous or diffuse-porous anatomy). A greater degree of incomplete rehydration, indicated by highest minimum TWD during TWD periods (70% increase of expected response for conifers and ring-porous species, 42% increase of expected response for diffuse-porous), together with warmer temperature (44% increase of expected response for all wood types) and stronger drought, prolonged the length of TWD periods. However, the recurrence of TWD periods only increased the length of the subsequent TWD period within the same year for diffuse-porous tree species (12% increase of expected response). Our results show that wood type and its associated functional traits influence tree responses to recurring stem dehydration periods. The greater sensitivity of diffuse-porous species to recurring TWD periods suggests that wood type anatomy may play an important role in determining how tree species cope with increasingly frequent summer droughts.

**Supplementary Information:**

The online version contains supplementary material available at https://doi.org/10.1007/s10342-026-01956-9.

## Introduction

Climate change has increased the occurrence of droughts and other disturbances in forest ecosystems worldwide (Seidl et al. 2017; Senf et al. 2020), leading to a global increase in drought-induced tree mortality (McDowell et al. 2022). Droughts can occur due to a lack of rain alone or in combination with increased temperature. Both are likely to become more frequent in Europe (Spinoni et al. 2018; Manning et al. 2019), exacerbating drought stress in forests. Drought stress can reduce tree productivity (Kannenberg et al. 2019), defence against pests and diseases (Stephenson et al. 2019), and increase the susceptibility to forest fires (Whitman et al. 2019). In addition to extreme droughts, the increased frequency of mild drought events can also significantly reduce the growth and the carbon sink strength of forests (Brzostek et al. 2014; Bose et al. 2025). The lowered yield and increased susceptibility to disturbances may cause significant economic losses to the forest industry, and a general reduction of ecosystem services, such as carbon storage (Thom and Seidl 2016).

Many studies on tree growth responses to drought stress use tree-ring width data with a yearly resolution (Lloret et al. 2011; Camarero et al. 2018), making it challenging to observe the seasonal behaviour of trees and the impact of frequent drought-stress periods within a growing season. Automated dendrometers enable a feasible way to record stem diameter changes at a high temporal resolution, and on a micrometre scale. There are two physiological processes behind the changes in stem diameter: firstly, irreversible expansion caused by the accumulation of new cells (wood and bark), and secondly, reversible expansion and contraction caused by the replenishment and depletion of stem water (Zweifel and Häsler 2001). At the sub-daily time scale, stems contract and expand following the atmospheric evaporative demand buffering the temporal imbalance between canopy transpiration and root water uptake. Consequently, stems often contract during the daytime (Zweifel et al. 2001; De Schepper et al. 2012; Steppe et al. 2015), when canopy transpiration exceeds root water uptake, and expand in the evening and nighttime, when vapour pressure deficit (VPD) decreases and water loss is minimal (Zweifel et al. 2021a, b; Peters et al. 2023; Feuer et al. 2025), resulting in the refilling of stem water pools. Thus, we can observe tree dehydration and rehydration dynamics on a sub-daily scale (Peters et al. 2023, 2025a), which can improve the mechanistic understanding of the impacts of drought-stress on trees (Sevanto 2025).

Tree water deficit (TWD), defined as the difference between the current stem radius and the preceding maximum radius (Zweifel et al. 2016), is caused by reversible stem contraction due to dehydration. It is related to the water content of the living tissues in the stem bark (Herzog et al. 1995), and is primarily driven by the evaporative demand of the atmosphere and the soil water status (Zweifel et al. 2005; Brito et al. 2017). Therefore, it is proposed as a good indicator of drought-stress in trees (Hinckley et al. 1978; Zweifel et al. 2005; Dietrich et al. 2018; Peters et al. 2025a). TWD can be calculated from the changes in stem diameter measurements after accounting for stem growth (Zweifel et al. 2016; Peters et al. 2025a). When the atmospheric evaporative demand increases and soil water availability decreases, trees may no longer be able to fully replenish their stem-water reserves and TWD remains positive (Zweifel et al. 2001). During these TWD (stem contraction) periods, very little or no growth is possible according to the zero-growth concept (Zweifel et al. 2016). During a prolonged drought, TWD can remain positive for several days, weeks or even months, indicating a continuous stress for a tree, thereby limiting temporal windows for growth. Furthermore, TWD dynamics are also linked to stomatal regulation, with insufficient nocturnal rehydration leading to diurnal stomatal closure (Peters et al. 2025b). This, in turn, can reduce carbon assimilation and may eventually lead to carbon depletion if stomata remain closed over extended periods (McDowell et al. 2008, 2022). Identifying the factors that prolong TWD periods is therefore essential for understanding the persistence of drought-induced stem dehydration and its potential consequences for tree-atmosphere gas exchange and stem growth.

Tree species differ in their phenology and therefore in the timing and duration of the growing season (Delpierre et al. 2016; Etzold et al. 2022; D’Orangeville et al. 2022). Moreover, tree responses to drought-stress are species-specific, according to their hydraulic strategy and architecture. Based on their wood anatomy, tree species can be divided into conifers, which have xylem consisting of tracheids, and broadleaved species with vessels, fibres and living parenchyma (Pfautsch 2016). Broadleaves are further divided into ring-porous species and diffuse-porous species, based on the distribution and size of their water-conducting elements, the xylem vessels (Zanne et al. 2010). These three wood types differ in their absolute water transport capacities, which depend partly on their belowground water uptake capacity (Walthert et al. 2024). The extent and reversibility of TWD are also closely linked to their xylem hydraulic properties. Especially in mature trees, xylem properties can influence the species’ capacity to rehydrate and therefore reduce TWD during the night, with observed differences between ring-porous, diffuse-porous, and coniferous species (Peters et al. 2023).

Recent years have seen a remarkable advancement in our understanding of TWD drivers and their relationships to physiological functions and properties (Zweifel et al. 2021a, b; Preisler et al. 2021; Salomón et al. 2022, 2025; Peters et al. 2023, 2025a, b; Ziegler et al. 2024; Salomón and Camarero 2025; Feuer et al. 2025). However, the length and recurrence of the TWD periods and how those may vary between the different wood types remain less studied. 

The duration of a TWD period reflects the period of negative water balance and thus also the phase during which a tree is unable to grow due to reduced water potentials and turgor pressures, for example in the cambium (Zweifel et al. 2016). Different tree species have different minimum water potentials that they can reach without suffering damage (Tyree and Zimmerman 2002, Choat et al. 2012). In combination with species-specific water transport conductivities (Tyree and Zimmerman 2002), this results in varying temporal patterns regarding whether and how quickly tree species recover from TWD periods. Consequently, the duration and intensity of TWD periods are directly related to the ability of tree species to cope with drought stress. In this study, we investigate whether the duration of individual TWD periods provides insight into the dynamics with which different tree species cope with dry periods and their rehydration. This approach is novel and differs from the standard method, which analyses the cumulative number of days with positive TWD over a growing season and thus does not consider the timing of TWD periods within a season. Our approach allows us to investigate whether the recurrence of TWD phases—i.e. the reappearance of a TWD phase following a recovery within the same growing season—influences subsequent TWD development and, for example, prolongs it. An understanding of how the duration of TWD phases differs between trees of different species, and how the response varies depending on the recurrence of water deficit phases and climatic conditions, would provide valuable information on the primary factors governing the seasonal water balance of trees.

Because response patterns among different wood types may not be apparent in site-level studies, analyses based on large-scale datasets encompassing multiple tree species and sites across diverse environmental conditions are needed. Here, we investigate whether different wood types, namely conifers, ring-porous, and diffuse-porous species, exhibit consistent responses to climatic conditions and to the recurrence of TWD periods within a growing season. To do this, we utilise a large European data set including several species within each wood type and sites across different environmental conditions, allowing us to identify wood-type-specific patterns at a continental scale. Our aim is to answer the following questions: (a) How does wood type influence the response of TWD period length to climatic conditions? and (b) Does the recurrence of TWD periods increase the TWD period length for the different wood types? We hypothesize that (a) warmer and drier conditions would increase the length of TWD periods for all wood types but in different magnitude with conifers having the strongest response, and (b) that TWD recurrence would further increase the length of TWD periods. To our knowledge, this is the first attempt to analyse the length of TWD periods, their recurrence and the effect of wood type on a large spatial (continental) scale.

## Materials and methods

### Data collection and processing

We used tree-specific point- and band dendrometer measurements from 132 monitoring plots across Europe, consisting of measurements of 766 trees from 29 species with a temporal resolution of 5 to 60 min (Online Resource Table S1). The dendrometers represented 10 different brands and 14 different models. The length of the measurement periods varied between sites with the longest being 16 years (2003–2018) and the shortest being one year. The data originated from the COST DenDrought2018 initiative (Salomón et al. 2022). Metadata of the sites are available in the Online Resource Table S1.

Each species was assigned to its respective wood type based on the literature (Online Resource Table 2). From each site, we used daily temperature data from the E-OBS dataset (Cornes et al. 2018) and Monthly Standardized Precipitation Evapotranspiration Index (SPEI) calculated from the SPEIbase (Vicente-Serrano et al. 2010; Beguería et al. 2010, 2014). We chose the E-OBS derived temperature instead of site measured temperatures to ensure the consistency of measurement conditions and the coverage of the whole time series. We rescaled both data sets with a 0.1-degree horizontal resolution to the site coordinates. The temperature was corrected for elevation by calculating the difference between the elevation of E-OBS and the actual site elevation, multiplying it by a fixed temperature lapse rate of 0.65° per 100 m (Stone and Carlson 1979), which was then added to the E-OBS temperature. VPD was calculated based on the E-OBS derived temperature and relative humidity (Cornes et al. 2018) using *plantecophys* R package version 1.4.6 (Duursma 2015). The distributions of mean and maximum temperature and mean SPEI during the analysed TWD periods are presented in the Online Resource Figures S1–S3. Afterwards, we standardised the temperature to a mean of zero and a standard deviation of one (z-transformation) prior to analyses to facilitate the comparison between the sites (Online Resource Figure S4).

**Fig. 1:**
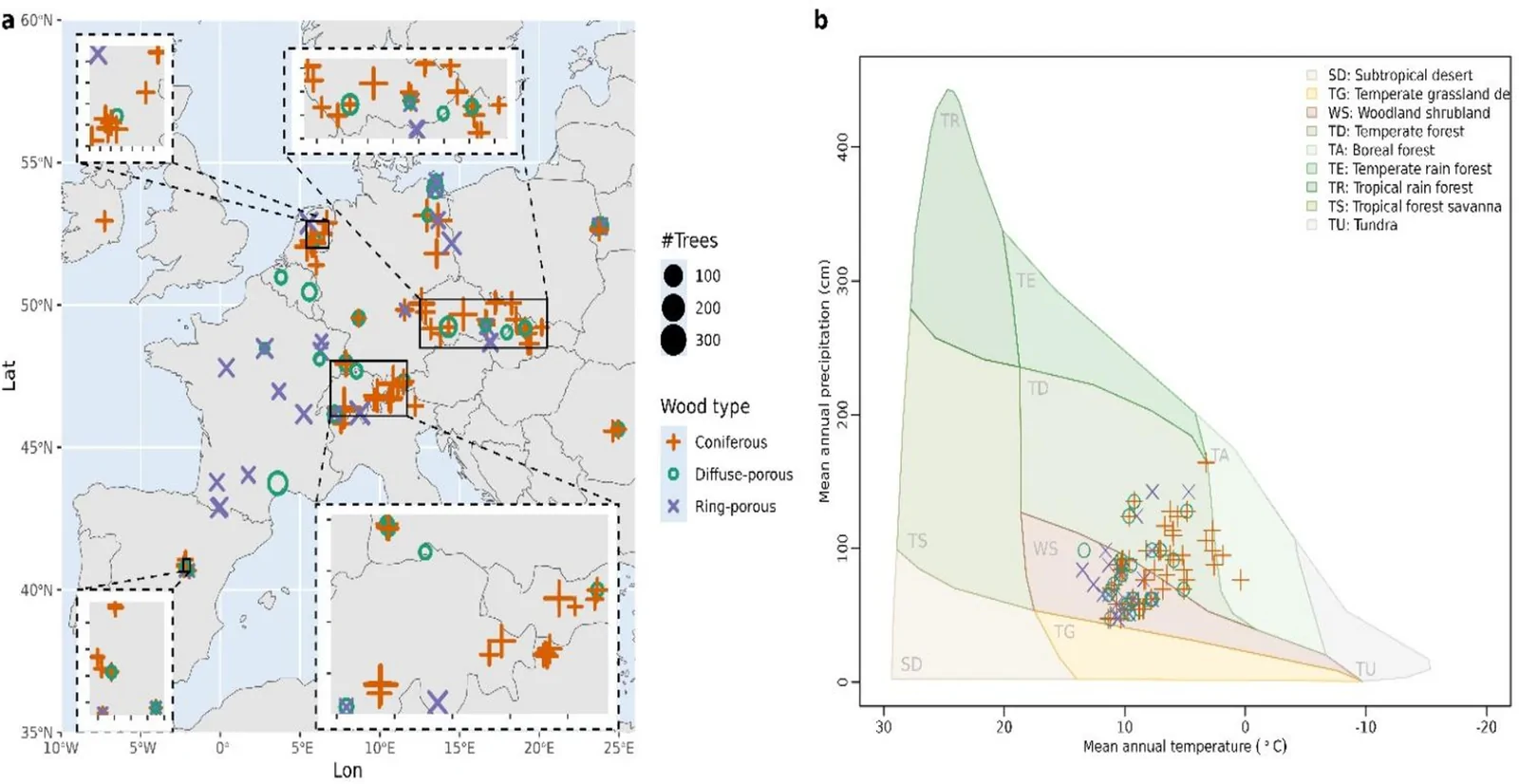
The geographical extent of the study as illustrated by (**a**) the location of sites in Europe, with size of the symbols indicating the number of trees per site, and (**b**) the occurrence of the study trees across biomes as proposed by Whittaker (1975) modified by Ricklefs (2008), and displayed as a function of mean annual temperature and mean annual precipitation. Different wood types are indicated by different symbols and colours, with an orange plus for coniferous species, a green circle for diffuse-porous species, and a purple X for ring-porous species

Stem diameter variation (stem ΔD) time series were processed with *treenetproc* R package version 0.1.4 (Haeni et al. 2020; Knüsel et al. 2021), which computed a growth- and a water-related component (TWD), following the zero-growth concept of Zweifel et al. (2016). The sub-hourly data was averaged into hourly values, and the temporal resolution of the dendrometer data being further analysed was based on hourly values, using the common time resolution of all available time series. All stem ΔD time series were visually checked, and time series with measurement errors such as artefactual jumps or seemingly dying trees with negative stem diameter trends over the entire season were excluded. This led to the removal of 31 trees from the data set. To ensure that the data only reflected TWD caused by dryness-related water stress and excluded freezing-thawing cycles, we limited the analysis to the summer (June, July and August), as many sites experienced freezing temperatures below zero during May and September. This led to the exclusion of 42 trees that did not experience TWD periods fulfilling the criteria from 23 sites and 11 countries. Therefore, the data used for the analysis consisted of 130 sites and 693 trees, including 387 coniferous (consisting of 10 species), 123 diffuse-porous (consisting of 9 species), and 183 ring-porous (consisting of 10 species) trees (Fig. 1a). The sites covered biomes across southern woodland shrublands, temperate and alpine (boreal) forests (Fig. 1b).

**Fig. 2:**
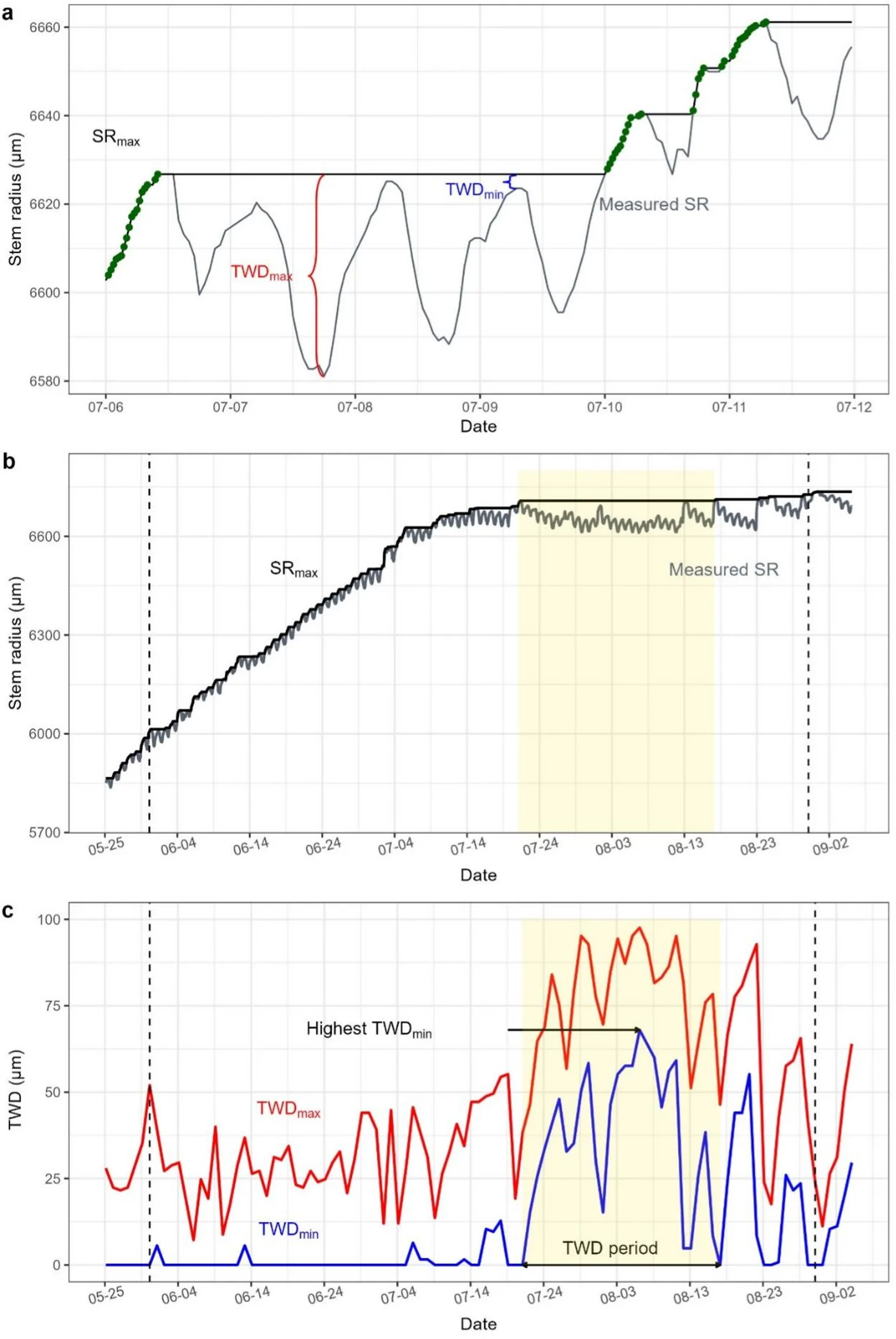
Illustrative example of dendrometer-derived variables: (**a**) dynamics of stem radius (SR, grey) and daily maximum stem radius (SR_max,_ black), occurrence of stem radius growth (green) and daily minimum (TWD_min_, blue) and maximum (TWD_max_, red) tree water deficit across a few days. (**b**) stem radius (SR, grey) and daily maximum stem radius (SR_max_, black) across summer. (**c**) daily minimum (TWD_min_, blue) and maximum (TWD_max_, red) tree water deficit across summer. The x-axis shows the date of the measurement period. The areas highlighted in light yellow in b and c indicate a TWD period (TWD > 0 for at least 120 h, i.e., five days). The dashed lines indicate the start and end of the summer season for the analysis of TWD periods. The black arrows point to the highest TWD_min_ of a TWD period, as a proxy of the TWD intensity. The figure is based on data from a beech tree at the Guenterstal site, Germany (see Online Resource Table S1)

In this study, we defined a TWD period as the interval during which TWD remained above zero for at least 120 h (equivalent to five 24-hour intervals) (Fig. 2). This period was chosen because short-term physiological responses (e.g., changes in stomatal conductance or osmotic adjustment across plant tissues) begin immediately with water shortage (Traversari et al. 2018; Ziegler et al. 2024), while other responses (e.g., embolism, cell wall collapse) may occur several days after drought sets in (Abe et al. 2003). This criterion helps to exclude very short periods that might not significantly affect tree physiology. To check the robustness of our results to the selected minimum duration of a TWD period, we conducted a sensitivity analysis using minimum durations of 72 and 168 h, during which TWD remained above zero. To describe the intensity of the TWD period, we calculated both daily TWD_min_ and TWD_max_ values. TWD_min_ generally occurs at night and represents the residual deficit after night-time rehydration; zero indicates recovery to the previous maximum stem radius, whereas positive values indicate incomplete recovery. TWD_max_ generally occurs during the day and combines this nocturnal residual deficit with additional diurnal shrinkage driven by transpiration and atmospheric evaporative demand (Fig. 2a). Therefore, the highest TWD_min_ during the TWD period describes the most insufficient level of recovery. An overview of the length and number of TWD periods per year, for the different wood types, is given in Online Resource Figure S5.

### Statistical analysis

Standardizing TWD values facilitates comparisons among different trees (Zweifel et al. 2005, 2007; Brinkmann et al. 2016). We standardised TWD for each tree with a z-score transformation (mean = 0 and standard deviation = 1). We aggregated the data by first calculating the daily minimum and maximum values for the standardised temperature and standardised TWD, and second calculating the minimum and maximum of the daily values of standardised temperature, SPEI, and standardised TWD for each detected TWD period, which in turn were used for model fitting.

To investigate the driving factors of TWD period length, we modelled the count of days using generalised linear mixed effects models (GLMM) with a log link, estimated by maximum likelihood estimation (Laplace approximation). Because the model used a log link, the fixed-effect coefficients were exponentiated and interpreted as incidence rate ratios (IRR), which represent proportional changes in the expected modelled response (here the length of TWD period) with associated predictors. We included a random effect for the individual tree nested within the measurement site, and a crossed random effect for the measurement year to account for spatial variation between sites and tree individuals, as well as for the temporal variation between years, respectively. Based on preliminary models (Online Resource Tables S3 and S4, Online Resource Figures S6 and S7), the fixed effects included in the final model were (1) highest TWD_min_ within the TWD period, (2) maximum temperature within the TWD period (from here on temperature), (3) minimum SPEI within the TWD period (from here on SPEI), (4) wood type, and (5) the recurrence of the TWD period within the summer season (e.g., the 1st, 2nd or n^th^ period of the summer, starting at 1), and the interactions amongst them (Online Resource Table S7). Among the TWD-derived variables, the highest TWD_min_ during each period was selected because it yielded the lowest AIC and is biologically meaningful as an indicator of the greatest deficit remaining after night-time rehydration. Because VPD was strongly correlated with temperature (Spearman *r* > 0.82), and replacing temperature or SPEI by VPD did not yield lower AIC values (Online Resource Table S4), VPD was excluded from the models. To test for potential differences in the response slope among wood types we used the *emtrends* function from the *emmeans* package in R (Lenth and Piaskowski 2026).

We initially fitted Poisson GLMMs and assessed the temporal autocorrelation of residuals, homoscedasticity and normality of the residuals, and overdispersion using the *DHARMa* R package (Hartig 2025). The model did not show temporal autocorrelation of residuals, but residual diagnostics indicated substantial overdispersion. To address this, we tested alternative specifications, including a Poisson model with an observation-level random effect, and a zero-inflated Poisson model (Harrison et al. 2018). Both approaches reduced but did not fully resolve overdispersion. Therefore, we adopted negative binomial GLMMs (nbinom2 family), which provided an appropriate fit for the data (Online Resource Figure S8). Model selection was based on AIC and residual diagnostics (Online Resource Table 5), and the final model was fitted using the *glmmTMB* package (Brooks et al. 2017). We used the *tidyverse* package for the data processing (Wickham et al. 2019) and the *ggeffects* package (Lüdecke 2018) for model illustration. All analyses were conducted in R (R Core Team 2025).

As the dataset was collected using 14 different dendrometer models (see Online Resource Table S1), we assessed the potential confounding effect of different sensor sensitivity by first modelling the TWD period length as a function of dendrometer model only, and then refitting the statistical model including dendrometer model as a fixed effect. We furthermore analysed a potential sampling bias: as many of the sites with ring-porous species and conifers were located in Mediterranean sites located south of 45° N (from Spain and the south of France), we tested whether the inclusion of these notably warmer and drier sites would influence the results. For this, we ran the model without sites below 45° latitude and compared the outcomes with those of the full model. In addition, we evaluated whether the recurrence of TWD periods was associated with seasonal timing by testing the correlation between TWD period recurrence and the calendar month in which the TWD period started separately for each wood type.

## Results

**Fig. 3:**
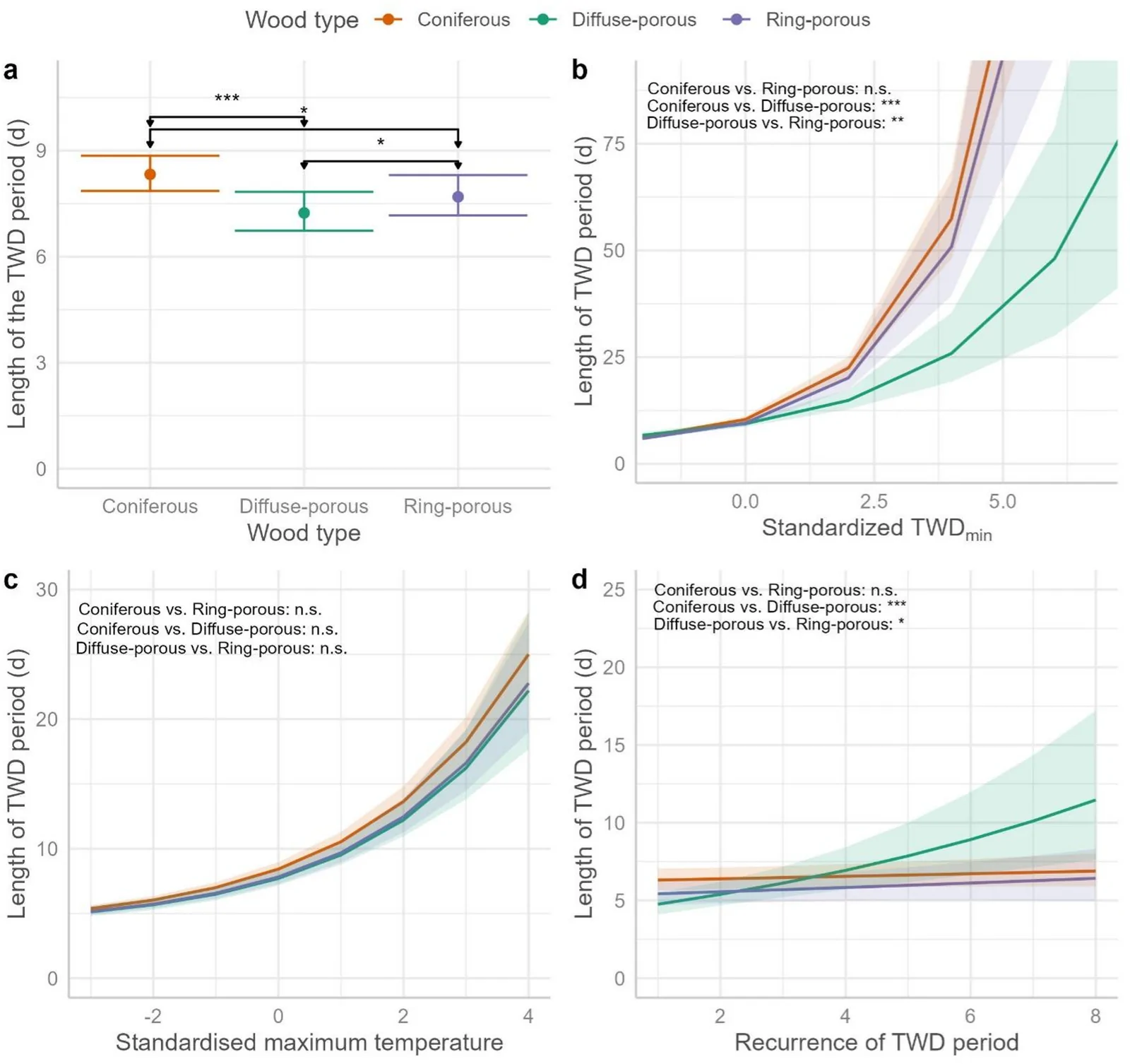
The predicted effects showing the predicted value and 95% confidence interval of (**a**) wood type, (**b**) TWD_min_, (**c**) temperature and (**d**) recurrence of TWD period within the season on the length of the TWD period. Colours indicate the wood type with orange = coniferous, green = diffuse-porous and purple = ring-porous. The significance signs indicate whether the pairwise comparison of the slopes was significant, with ***, **, and * indicating the *p*-values below 0.001, 0.01, and 0.05 respectively. Note that the y-axis scales differ in each panel

The length of the TWD period was best explained by the highest TWD_min_ during the TWD period, followed by temperature, the wood type, and the interaction between temperature and SPEI (Online Resource Table S7). Under reference conditions (i.e., when model predictors are 0), the expected length of a single TWD period for conifers was 7.46 days (95% CI: 7.07–7.91; *p* < 0.01). For ring-porous species, it was 16% lower than that of conifers (IRR ≈ 0.84; *p* < 0.05), while for diffuse-porous species, it was 33% lower relative to conifers (IRR ≈ 0.67; *p* < 0.01) (Fig. 3a, Online Resource Table S7) and 26% lower than for ring-porous species (IRR ≈ 1.26; *p* < 0.05). Note that IRRs represent proportional differences in the expected response and should not be interpreted as additive or subtractive changes relative to the reference baseline. The interaction between standardized TWD_min_ and wood type was significant (*p* < 0.01; Fig. 3b): a unit increase in standardized TWD_min_ increased the expected response of TWD period length by 70% for conifers and ring-porous species (IRR ≈ 1.70), and by 42% for diffuse-porous species (IRR ≈ 1.42). Thus, a higher standardized TWD_min_ resulted in significantly longer TWD periods for coniferous and ring-porous species compared to diffuse-porous ones (*p* < 0.01; Fig. 3b), while the difference between conifers and ring-porous species was not significant.

**Fig. 4:**
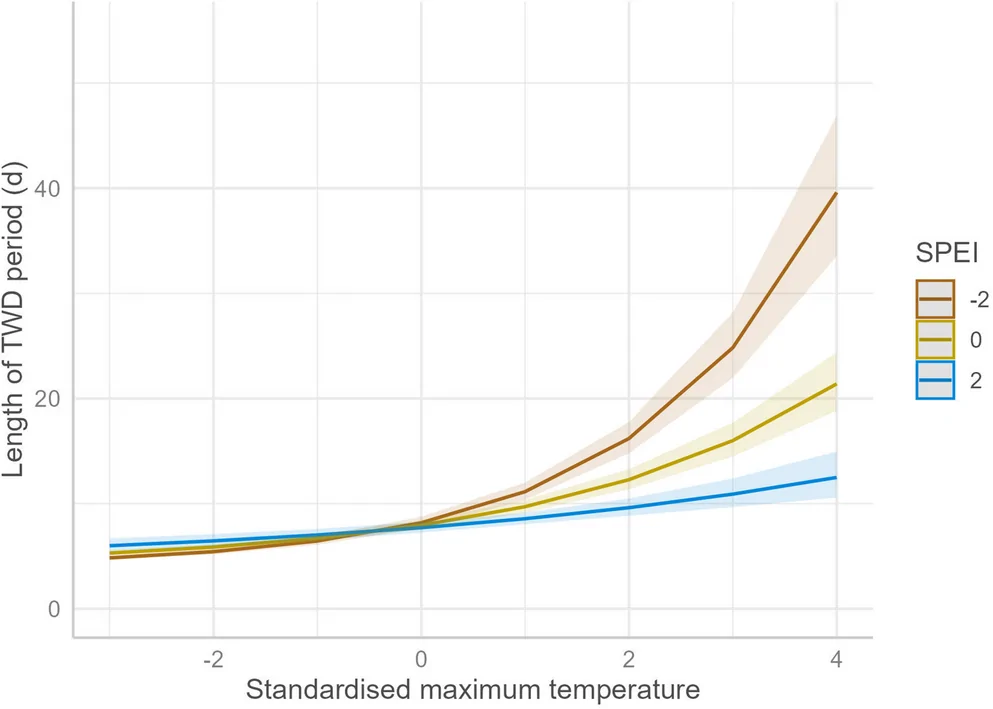
The interaction effect of standardised maximum daily temperature and SPEI on the length of the TWD period for all wood types combined. The length of TWD periods was greatest under conditions of high temperature and low SPEI, and the temperature × SPEI interaction was significant (*p* < 0.001). Colours indicate the magnitude of SPEI with reddish brown being very dry (SPEI = − 2), light brown average condition (SPEI = 0) and blue very wet (SPEI = 2)

A unit increase of standardized temperature increased the expected TWD period length on average by 44% (*p* < 0.01; IRR ≈ 1.44; Fig. 3c), with no significant difference between wood types. While the SPEI value alone was not statistically significant, its interaction with temperature was negative and significant (*p* < 0.001). This indicates that with wetter conditions (i.e., higher SPEI), the effect of temperature on the TWD length is lower (Fig. 4) and vice versa, denoting that dry and hot conditions increase the length of TWD periods.

The recurrence of TWD periods within the summer season did not influence the length of the TWD period overall (*p* > 0.05). However, the interaction between wood type and the recurrence of the TWD period was significant, with diffuse-porous species experiencing longer TWD periods with an increasing recurrence of TWD periods (IRR ≈ 1.12; *p* < 0.01; Fig. 3d). This effect was especially evident under hotter and drier conditions (Online Resource Figure S9). Overall, the fixed factors explained 40.1% of the variation in TWD period length, and the full model explained 53.5%.

The sensitivity analysis using minimum TWD period durations of 72 and 168 h showed highly consistent results with those obtained using the main 120 h threshold (Online Resource Table S8, Online Resource Figure S10). As expected, the 72-h threshold resulted in more TWD periods within a season (maximum number of periods = 12, median number of periods = 3) than the 120-h (maximum number of periods = 8, median number of periods = 2), and 168-h (maximum number of periods = 6, median number of periods = 2) thresholds. Despite this difference, model estimates remained largely unchanged.

When modelling dendrometer model alone as an explanatory variable for a TWD period length, only one model (TOMST) used in four sites and on 30 trees had significantly shorter TWD period length (*p* < 0.05). The dendrometer model-related variation did not meaningfully affect the results: when the dendrometer model was included as a fixed effect in the full model, the change in model estimates was small: including the dendrometer model increased the estimate of the intercept by 0.12 and the upper 95% CI limit by 0.26 while the lower boundary remained the same. Including the dendrometer model increased the estimates of wood types by 0.05 or less, while the 95% CI were almost identical. Estimates of other variables changed only by 0.01. Similarly, when testing the effect of potential sampling bias caused by the sites in the Mediterranean region (below 45° latitude), we observed no notable changes in model estimates: the greatest change in model estimates was 0.06 and 0.08 in 95% CIs. Finally, the correlation between the recurrence of TWD periods and the calendar month of TWD onset varied among wood types, ranging from weak in diffuse-porous species (*r* = 0.34) to moderate in ring-porous (*r* = 0.58) and coniferous species (*r* = 0.55), indicating that recurrence and seasonal timing were only partially associated.

## Discussion

We analysed how temperature, water availability and evaporative demand (SPEI), TWD intensity, wood type and the recurrence of TWD periods within the summer season affected the length of TWD periods based on a European-wide dendrometer dataset. Our results showed that a higher TWD_min_, i.e., lower level of rehydration, during a TWD period increased its length, but the reaction differed between the wood types with conifers and ring-porous species having longer TWD periods than diffuse-porous species. The results furthermore supported our first hypothesis that warmer and drier conditions do increase the length of TWD periods for all wood types. The length of TWD periods did increase with period recurrence for diffuse-porous species but not for conifers and ring-porous species, which contrasted in part with our second hypothesis.

### The effect of stem contraction and climatic factors on the length of a TWD period

The highest TWD_min_ during the TWD period had the greatest effect on the length of the period when compared to the other explanatory variables for all wood types, but with less-pronounced effects on diffuse-porous species (Fig. 3b). The TWD_min_ reflects the capacity of trees to rehydrate their stem water reserves overnight, with high TWD_min_ values indicating limited levels of rehydration and therefore a higher level of drought stress on a daily and seasonal temporal scale. Thus, the results suggest that the longer TWD periods can be also more severe in terms of stem contraction than shorter ones. The more the stem contracts, the more stem-water reserves are depleted, and this advances with the depletion of soil water reserves (e.g., Peters et al. 2023).

Among the assessed climatic variables, the maximum temperature during a TWD period best explained its length, with a uniform response from all wood types (Fig. 3c; Online Resource Table S6). This denotes the primary role of atmospheric drivers as causal factors for stem contraction and tree stress. Extreme temperatures alone cause heat stress and may lead to decreased growth, foliar damage and changes in resource allocation (Teskey et al. 2015; Still et al. 2023). The effect of air temperature was exacerbated by drier climatic conditions as denoted by the interaction between SPEI and temperature (Fig. 4). Lower SPEI values reflect a climatic water deficit, which may reduce soil water availability and consequently constrain root water uptake, and thus stem rehydration. These results add to the evidence provided by previous studies, where TWD intensity was shown to depend on both the soil water availability and evaporative demand (Zweifel et al. 2005; Brinkmann et al. 2016).

The consequence of the length and recurrence of TWD periods on tree stem growth depends on the timing of the TWD periods, mainly whether and when they occur during the time of wood formation (Lempereur et al. 2015; Etzold et al. 2022; Lauriks et al. 2022; Krejza et al. 2022). Tree stem diameter growth takes place in relatively short time windows (Steppe et al. 2015; Etzold et al. 2022) and is highly sensitive to water deficits (Zweifel et al. 2016). This is particularly relevant for species in drought-prone regions (Lempereur et al. 2015; Salomón and Camarero 2025). Therefore, long or frequently occurring TWD periods during the growing season commonly lead to major reductions in annual stem growth. However, if the adverse environmental conditions take place after or before tree stem diameter growth has peaked at around the summer solstice, the consequences in terms of cumulative annual growth are not necessarily severe, at least during the same growing season (D’Orangeville et al. 2018; Etzold et al. 2022). For example, the 2018 summer heatwave was not as detrimental overall to tree stem radial growth as feared, because it took place substantially after the main growth season in most sites (Salomón et al. 2022). Nevertheless, regional growth losses could have been severe during (Krejza et al. 2021) or after the heatwave due to legacy effects (Walthert et al. 2021). In addition, stem biomass growth lags behind stem diameter growth (Cuny et al. 2015), and might therefore show different seasonal sensitivity to drought.

### The effect of wood type on the length of a TWD period

The three wood types showed different responses to TWD intensity and recurrence, which influenced the predicted TWD period length. As expected, conifers experienced slightly longer periods than ring- or diffuse-porous species (Fig. 3a, Online Resource Figure S5) because conifers have large internal water storage in combination with a low water conductance of the wood (Peters et al. 2023). With a similar data set, Salomón et al. (2022) observed that conifers had less capacity to rehydrate overnight during the 2018 heatwave. Furthermore, the limited access to deep soil water (Walthert et al. 2024) may limit the rate at which coniferous trees refill their stem water reserves, which might lead to prolonged TWD periods compared to broadleaved trees that can more rapidly restore their stem water reserves. Previous studies have also shown that coniferous trees have the highest standardized pre-dawn TWD (which corresponds to our TWD_min_) in response to a given decrease in soil water potential (Peters et al. 2023) when compared with ring- and diffuse-porous species. Unexpectedly, the response of ring-porous species to increased TWD_min_ was similar to that of conifers. This pattern remained also when the drier and warmer sites (below 45° N) were excluded from the data set to test the robustness of the results, so that most TWD periods occurred at common European temperatures below 30° C (Online Resource Figure S2). Peters et al. (2023) found that ring-porous *Quercus petraea* had the lowest normalized pre-dawn TWD in response to decreases in soil water potential compared to conifers and diffuse-porous species. This apparently contrasting finding could indicate that some ring-porous species prioritise limiting the depletion of stem water reserves under drought stress. Resolving the mechanisms underlying these patterns will require species-specific analyses with a more detailed dataset than was available here, integrating rooting depth, stomatal regulation and xylem vulnerability to embolism, which jointly regulate stem water depletion and refilling.

The recurrence of TWD periods within a season resulted in wood type-specific responses with a significant increase in the length of TWD periods for diffuse-porous species, especially with warmer temperature and lower SPEI values (Fig. 4d, Online Resource Figure S1, S3 and, S9). This pattern may reflect cumulative impairment of stem hydraulic integrity, progressively constraining stem rehydration. However, and contrary to our hypothesis, TWD length in coniferous and ring-porous broadleaved trees did not respond to TWD recurrence. For conifer species, the lower capacity for rehydration (Salomón et al. 2022; Peters et al. 2023) likely contributes to their lack of clear response to recurrent TWD periods, as the low overnight rehydration leads to longer TWD periods in general and changes with recurring periods are less prominent. For ring-porous species, we expected to see a greater sensitivity to recurrent TWD periods, due to predominant embolism of large early-wood vessels (Lobo et al. 2018), which can affect recovery capacity after recurrent droughts. The observed difference between ring- and diffuse-porous species may relate to their wood phenology and potential consequences on their stomatal regulation. Diffuse-porous species have a delayed wood phenology relative to ring-porous species, as earlywood vessels in diffuse-porous species are formed after leaf flush (Barbaroux and Breda 2002). Therefore, their peak growth takes place later in the season under which more severe water deficits may occur more frequently (D’Orangeville et al. 2022). Thus, some diffuse-porous species might have less conservative stomatal behaviour later in the season to maintain carbon assimilation and stem growth, even at the risk of stem hydraulic failure and dehydration (Von Allmen et al. 2015; Ouyang et al. 2017). However, the correlation between TWD period recurrence and the calendar month of TWD onset was relatively weak for diffuse-porous species (*r* = 0.34), indicating that recurrence and seasonal timing represented only partly overlapping sources of variation. This suggests that the observed recurrence effect is unlikely to be explained solely by the tendency of diffuse-porous species to experience TWD periods later in the growing season. Instead, repeated TWD periods may have cumulative effects on stem water relations that extend beyond seasonal progression alone. Eventually, this higher sensitivity may contribute to the observed anomalies in community-level mortality rates associated with drought in diffuse-porous species (Anderegg et al. 2016). Nevertheless, it should be noted that the dataset was unbalanced regarding the recurrence of TWD periods, with most trees experiencing less than four TWD periods during the summer season. Consequently, predictions of the TWD length at higher recurrence levels were more uncertain.

In addition to wood type, there are many tree traits influencing stem dehydration, e.g., stomatal regulation, resistance to embolism or rooting depth (Nardini et al. 2016; D’Orangeville et al. 2022). Therefore, species with the same wood anatomy may have very different responses to drought stress (Güney et al. 2020) according to their water use economy and physiological adaptations to local conditions. For example, the diffuse-porous *Fagus sylvatica* is highly vulnerable to tree water deficits (Walthert et al. 2021), whereas another diffuse-porous species, *Sorbus torminalis*, well-adapted to warm climates (Thomas 2017), is more resistant to periods of water deficit (Peters et al. 2025a). Similarly, the specific site conditions may influence the tree species responses to drought stress due to differences in e.g., soil type and slope of the site. Our analysis on site-specific responses was limited due to the data availability on site characteristics. However, we could include more trees, species, sites and environmental conditions than would have been possible in a more site-specific study. This allowed us to identify broad wood-type-specific patterns, while still counting the site-specific variability in responses with mixed-effect modelling. Further studies could test potential differences among hydraulic strategies in different site conditions to better understand the full tree response to water deficit and whether the reaction of different tree species to changes in TWD is similar. Promising new approaches are shown in e.g., Peters et al. (2025a), who validated the use of a novel TWD index, with measurements of leaf water potential, stomatal conductance, and critical hydraulic thresholds, and Salomón et al. (2025), who were able to report differences in the sequence of stomatal closure, stem dehydration and xylem embolism across species.

## Conclusion

In this study, we used a European dataset encompassing diverse tree species and environmental conditions to investigate the factors controlling the length of TWD periods. The strongest predictor of TWD period length was the highest TWD_min_, indicating that the severity of stem dehydration influences the duration of tree stress. In addition, hotter and drier conditions, reflected by the interaction between high temperature and low SPEI, substantially prolonged TWD periods, highlighting the combined importance of atmospheric drivers in regulating tree water status.

Wood type influenced both the baseline length of TWD periods and their response to stem dehydration. Coniferous and ring-porous species showed stronger increases in TWD period length with increasing TWD_min_ than diffuse-porous species, whereas diffuse-porous species responded more strongly to the recurrence of TWD periods. These findings suggest that wood type may play an important role in determining how tree species respond to increasingly hot and dry summers. Determining the exact mechanisms behind TWD period length requires more specific studies. As droughts intensify under climate change, wood-type-specific differences in stem dehydration and recovery may influence forest growth, resilience and ecosystem functioning across European forests.

## Supplementary Information

Below is the link to the electronic supplementary material.

Supplementary Material 1

## Data Availability

Data used for modelling is available in Zenodo repository: https://doi.org/10.5281/zenodo.22012008. The full dataset is available from the authors upon a reasonable request.

## Code availability

No code was developed for this analysis.
