# Supplementary material for: Wood type influences tree responses to recurring hot and dry summer tree water deficits across European forests
Source: Eur J For Res. 2026 Oct 9;145(6):120. doi: 10.1007/s10342-026-01956-9 (PMC13652923; doi:10.1007/s10342-026-01956-9)
Supplement: Supplementary file 1 [file 10342_2026_1956_MOESM1_ESM.docx]

Online Resources

Online Resource Table S1**.** Metadata on sites and the measured trees. The coordinates for sites from Zin et al. are approximation due to the current geopolitical state.

| **Site** | **#trees** | **Species** | **Lat** | **Lon** | **Altitude (masl)** | **Measurement start date** | **Measurement end date** | **Dendrometer model** |
| --- | --- | --- | --- | --- | --- | --- | --- | --- |
| Bär.Mayr_Absamer.Vorberg_1559145547 | 9 | *Fagus sylvatica, Picea abies, Pinus sylvestris* | 47.3149 | 11.5112 | 875 | 01/04/2016 | 22/03/2019 | EMS Brno DRL26 |
| Cada_CZ_J01_1559055028 | 4 | *Picea abies* | 50.0754 | 17.2507 | 1209.92 | 13/11/2013 | 25/04/2019 | EMS Brno DRL26 |
| Cada_CZ_J08_1559055689 | 4 | *Picea abies* | 50.064 | 18.2577 | 185 | 14/11/2013 | 25/04/2019 | EMS Brno DRL26 |
| Cada_CZ_SB3_1559056260 | 4 | *Picea abies* | 48.9923 | 13.8213 | 1266.88 | 16/11/2013 | 14/05/2019 | EMS Brno DRL26 |
| Cada_CZ_SJ8_1559056710 | 4 | *Picea abies* | 49.1674 | 13.1888 | 1250.4 | 18/11/2013 | 14/05/2019 | EMS Brno DRL26 |
| Carraro_IT.SC_1558624837 | 5 | *Picea abies, Pinus sylvestris, Larix decidua* | 46.4501 | 12.2168 | 1000 | 01/01/2018 | 31/12/2018 | Midori LP-20F |
| Cremonese_IT.Trf_1558973752 | 3 | *Larix decidua* | 45.82306 | 7.56083 | 2160 | 25/05/2017 | 25/11/2018 | UMS D6 |
| Delpierre.Berveiller.Dufrêne_FRFon_1559141157 | 12 | *Carpinus betulus, Quercus petraea* | 48.47634 | 2.780055 | 92 | 08/08/2012 | 01/01/2019 | University of Oulu, ELPA98 |
| Delpierre.Cecchini_CHS01_1560497720 | 10 | *Quercus petraea* | 46.1708 | 5.238 | 260 | 31/01/2017 | 13/03/2019 | EMS Brno DRL26 |
| Delpierre.Cecchini_CHS58_1560497349 | 10 | *Quercus petraea* | 46.9696 | 3.66 | 270 | 28/02/2017 | 06/02/2019 | EMS Brno DRL26 |
| Delpierre.Cecchini_CHS72_1560437766 | 9 | *Quercus petraea* | 47.7955 | 0.3796 | 170 | 13/03/2017 | 13/02/2019 | EMS Brno DRL26 |
| Delpierre.Cecchini_CHS81_1560498069 | 5 | *Quercus petraea* | 44.04 | 1.75 | 300 | 26/02/2016 | 17/04/2019 | EMS Brno DRL26 |
| Delpierre.Delzon_Cheze_1560498976 | 10 | *Quercus petraea* | 42.9167 | -0.0333 | 803 | 25/01/2016 | 21/02/2019 | EMS Brno DRL26 |
| Delpierre.Delzon_Laveyron_1560498477 | 9 | *Quercus petraea* | 43.77611 | -0.2167 | 131 | 27/01/2016 | 12/03/2019 | EMS Brno DRL26 |
| Delpierre.Delzon_Peguere_1560500146 | 8 | *Quercus petraea* | 42.8667 | -0.1167 | 1630 | 26/01/2016 | 04/06/2019 | EMS Brno DRL26 |
| Delpierre.Lebourgeois_Charmes_1560436823 | 10 | *Quercus petraea* | 48.3725 | 6.293333 | 280 | 09/02/2017 | 07/03/2019 | EMS Brno DRL26 |
| Ehekircher_Schmellenhof_1559046878 | 5 | *Picea abies* | 49.826 | 11.5713 | 510 | 12/05/2017 | 28/09/2018 | Megatron MM® 10-11 |
| Ehekircher_Schmellenhof_1559048617 | 6 | *Pinus nigra* | 49.8262 | 11.5686 | 508 | 12/05/2017 | 06/11/2018 | Megatron MM® 10-11 |
| Ehekircher_Schmellenhof_1559049762 | 5 | *Quercus robur* | 49.8241 | 11.5758 | 512 | 12/05/2017 | 06/11/2018 | Megatron MM® 10-11 |
| Ehekircher_Weinheim_1559050429 | 3 | *Corylus colurna* | 49.541 | 8.6842 | 155 | 14/04/2017 | 12/02/2019 | Megatron MM® 10-11 |
| Ehekircher_Weinheim_1559051061 | 7 | *Juglans nigra* | 49.541 | 8.677 | 170 | 14/04/2017 | 24/01/2019 | Megatron MM® 10-11 |
| Ehekircher_Weinheim_1559051567 | 5 | *Metasequoia glyptostroboides* | 49.5406 | 8.677 | 190 | 14/04/2017 | 18/01/2019 | Megatron MM® 10-11 |
| Fonti_LTAL_N08_1558171652 | 9 | *Picea abies, Larix decidua* | 46.3026 | 7.7411 | 804 | 30/10/2007 | 01/01/2019 | Ecomatik DR |
| Fonti_LTAL_N13_1558174487 | 9 | *Picea abies, Larix decidua* | 46.3918 | 7.7613 | 1361 | 23/10/2006 | 01/01/2019 | Ecomatik DR and DC |
| Fonti_LTAL_N13W_1558202227 | 6 | *Picea abies, Larix decidua* | 46.3934 | 7.7639 | 1321 | 23/10/2006 | 01/01/2019 | Ecomatik DR |
| Fonti_LTAL_N16_1558180015 | 8 | *Picea abies, Larix decidua* | 46.3871 | 7.7643 | 1634 | 23/10/2006 | 01/01/2019 | Ecomatik DR and DC |
| Fonti_LTAL_N19_1558180434 | 8 | *Picea abies, Larix decidua* | 46.3869 | 7.7738 | 1961 | 23/10/2006 | 01/01/2019 | Ecomatik DR and DC |
| Fonti_LTAL_N22_1558180811 | 3 | *Larix decidua* | 46.3812 | 7.7728 | 2182 | 23/10/2006 | 01/01/2019 | Ecomatik DR and DC |
| Fonti_LTAL_S16_1558176718 | 10 | *Picea abies, Larix decidua* | 46.3972 | 7.7554 | 1670 | 23/10/2006 | 01/01/2019 | Ecomatik DR and DC |
| Fonti_LTAL_S19_1558177687 | 11 | *Picea abies, Larix decidua* | 46.3967 | 7.7459 | 1928 | 23/10/2006 | 01/01/2019 | Ecomatik DR and DC |
| Fonti_LTAL_S22_1558202465 | 5 | *Larix decidua* | 46.3996 | 7.7426 | 2104 | 23/10/2006 | 01/01/2019 | Ecomatik DR and DC |
| Forner.Valladares_Huertahernando.Plot.H_1561985218 | 4 | *Juniperus thurifera* | 40.8276 | -2.2749 | 1134 | 06/10/2017 | 31/12/2018 | EMS Brno DR26 |
| Forner.Valladares_Huertahernando.Plot.I_1561986402 | 4 | *Juniperus thurifera* | 40.8257 | -2.2769 | 1140 | 01/01/2017 | 31/12/2018 | EMS Brno DR26 |
| Forner.Valladares_Huertahernando.Plot.J_1561988479 | 3 | *Juniperus thurifera* | 40.8266 | -2.2786 | 1147 | 03/10/2017 | 31/12/2018 | EMS Brno DR26 |
| Forner.Valladares_Maranchon.Plot.C_1561976943 | 4 | *Juniperus thurifera* | 41.0581 | -2.1931 | 1343 | 04/10/2017 | 31/12/2018 | EMS Brno DRL26 |
| Forner.Valladares_Maranchon.plot_A_1561943218 | 2 | *Juniperus thurifera* | 41.0657 | -2.1963 | 1334 | 04/10/2017 | 31/12/2018 | EMS Brno EMS51 |
| Forner.Valladares_Ribarredonda.Plot.N_1561991369 | 3 | *Juniperus thurifera* | 40.8717 | -2.303 | 1034 | 03/10/2017 | 31/12/2018 | EMS Brno DR26 |
| Forner.Valladares_Ribarredonda.Plot.O_1561989248 | 4 | *Juniperus thurifera* | 40.8705 | -2.3012 | 1049 | 03/10/2017 | 31/12/2018 | EMS Brno DR26 |
| Forner.Valladares_Ribarredonda.Plot.Q_1561989812 | 3 | *Juniperus thurifera* | 40.8709 | -2.2995 | 1036 | 03/10/2017 | 31/12/2018 | EMS Brno DR26 |
| Ganthaler.Mayr_Patscherkofel_1560238648 | 6 | *Picea abies* | 47.22161 | 11.46767 | 1626.48 | 01/05/2017 | 30/04/2019 | EMS Brno DRL26 |
| Ganthaler.Mayr_Praxmar_1559147741 | 3 | *Picea abies* | 47.1525 | 11.1266 | 1895.76 | 01/05/2017 | 30/04/2019 | EMS Brno DRL26 |
| Hentschel.Hentschel_GER_BB_1203_1559563452 | 4 | *Pinus sylvestris* | 52.9736 | 13.6439 | 70 | 05/04/2008 | 31/12/2018 | UMS D4 |
| Hentschel_GER_BB_1202_1558711520 | 4 | *Pinus sylvestris* | 53.1347 | 12.965 | 79 | 01/04/2018 | 30/09/2018 | UMS D4 |
| Hentschel_GER_BB_1202_1559563185 | 4 | *Pinus sylvestris* | 53.1347 | 12.965 | 79 | 04/04/2008 | 31/12/2018 | UMS D4 |
| Hentschel_GER_BB_1205_1559563672 | 3 | *Pinus sylvestris* | 51.7983 | 13.5639 | 133 | 31/12/2009 | 31/12/2018 | UMS D4 |
| Hentschel_GER_BB_1207_1559563846 | 4 | *Fagus sylvatica* | 53.1533 | 12.9883 | 90 | 31/12/2016 | 31/12/2018 | UMS D4 |
| Hentschel_GER_BB_1208_1559564073 | 4 | *Quercus petraea* | 52.165 | 14.4975 | 118 | 13/03/2009 | 31/12/2018 | UMS D4 |
| Hentschel_GER_BB_1209_1559564327 | 4 | *Quercus petraea* | 52.98 | 13.65 | 70 | 31/12/2014 | 31/12/2018 | UMS D4 |
| Janda_SK_JAK_053_1559058930 | 4 | *Picea abies* | 49.0086 | 19.198 | 1303.99 | 22/03/2014 | 08/08/2018 | EMS Brno DRL26 |
| Janda_SK_JAK_071_1559059423 | 4 | *Picea abies* | 49.0098 | 19.1991 | 1263.71 | 22/03/2014 | 08/08/2018 | EMS Brno DRL26 |
| Jezik.Blazenec.Ditmarova.Strelcova_Predna.Polana_1560257582 | 3 | *Picea abies* | 48.6296 | 19.4621 | 1350 | 31/05/2006 | 26/10/2006 | EMS Brno DRL26 |
| Jezik.Blazenec.Jakus_Ocova_1560763196 | 8 | *Picea abies* | 48.6296 | 19.3049 | 535 | 11/04/2017 | 28/11/2018 | EMS Brno DRL26 |
| Jezik.Ditmarova.Blazenec.Jakus.Strelcova_Predna.Polana_1560326951 | 5 | *Picea abies* | 48.6296 | 19.4621 | 1350 | 15/03/2017 | 15/11/2018 | EMS Brno DRL26 |
| Knüsel.Conedera_Avegno.01_1558705965 | 7 | *Ailanthus altissima, Castanea sativa* | 46.21219 | 8.745667 | 320 | 12/03/2013 | 24/05/2019 | Natkon ZN11-T-WP |
| Knüsel.Conedera_Avegno.02_1558704843 | 2 | *Ailanthus altissima* | 46.21417 | 8.739722 | 340 | 08/04/2014 | 21/05/2019 | Natkon ZN11-T-WP |
| Krejza.Svetlík.Bellan_ES1000_1558962361 | 3 | *Picea abies* | 50.11777 | 17.24704 | 995 | 09/03/2016 | 12/12/2018 | EMS Brno DRL26 |
| Krejza.Svetlík.Bellan_Habruvka_1558947620 | 3 | *Picea abies* | 49.3111 | 16.71253 | 489 | 09/03/2016 | 13/12/2018 | EMS Brno DRL26 |
| Krejza.Svetlík.Bellan_Rajec_old_1558960237 | 3 | *Picea abies* | 49.4437 | 16.69649 | 625 | 10/03/2017 | 31/12/2018 | EMS Brno DRL26 |
| Krejza.Svetlík.Bellan_Rajec_young_1558959567 | 3 | *Picea abies* | 49.44571 | 16.69711 | 622 | 17/03/2017 | 28/02/2019 | EMS Brno DRL26 |
| Krejza.Svetlík.Bellan_Rovna_1559119345 | 3 | *Picea abies* | 49.48483 | 16.61466 | 381 | 13/03/2016 | 13/12/2018 | EMS Brno DRL26 |
| Krejza.Svetlík.Bellan_Vidly_1558949728 | 3 | *Picea abies* | 50.10638 | 17.26714 | 798 | 09/03/2016 | 12/12/2018 | EMS Brno DRL26 |
| Krejza_Bily.Kriz_1558365087 | 9 | *Picea abies* | 49.50206 | 18.53682 | 875 | 19/03/2016 | 20/12/2018 | EMS Brno DRL26 |
| Krejza_Stitna.nad.Vlari_1558515130 | 7 | *Fagus sylvatica* | 49.03594 | 17.96986 | 550 | 01/11/2017 | 04/01/2019 | EMS Brno DRL26 |
| Limousin.Ourcival_FR.Pue_1560782777 | 11 | *Quercus ilex* | 43.7415 | 3.5963 | 270 | 06/06/2003 | 01/01/2019 | University of Oulu, ELPA98 |
| Matula_Plot.24_1559517984 | 6 | *Quercus faginea, Pinus nigra, Pinus sylvestris, Quercus ilex* | 40.67883 | -1.94953 | 1369 | 20/10/2017 | 30/11/2018 | TOMST |
| ‍Matula_SPA.1_1560266640 | 4 | *Pinus sylvestris, Quercus faginea* | 40.659185 | -2.27042 | 1294 | 27/10/2017 | 31/12/2018 | TOMST |
| Matula_SPA.36_1560250439 | 6 | *Pinus nigra, Quercus ilex* | 40.81411 | -2.21761 | 1211 | 27/10/2017 | 31/12/2018 | TOMST |
| Mikolás_SK_SRA_008_1_1559061737 | 4 | *Abies alba, Picea abies, Fagus sylvatica* | 49.1899 | 19.1095 | 978.58 | 18/03/2015 | 12/04/2019 | EMS Brno DRL26 |
| Mikolás_SK_SRA_010_2_1559062328 | 5 | *Abies alba, Picea abies, Fagus sylvatica* | 49.1864 | 19.1083 | 954.87 | 18/03/2015 | 12/04/2019 | EMS Brno DRL26 |
| Nabuurs.Lerink_De.Heul_1559309341 | 5 | *Pseudotsuga menziesii* | 52.04887 | 5.44303 | 6 | 21/02/2008 | 31/12/2018 | EMS Brno DRL26 |
| Nabuurs.Lerink_Leesten_1559641683 | 5 | *Pseudotsuga menziesii* | 52.15662 | 5.90815 | 70 | 21/02/2008 | 31/12/2018 | EMS Brno DRL26 |
| Nabuurs.Lerink_Loobos_1559289159 | 4 | *Pinus sylvestris* | 52.16678 | 5.74385 | 22 | 01/03/2008 | 31/12/2018 | EMS Brno DRL26 |
| Nabuurs.Lerink_Motketel_1559315009 | 1 | *Fagus sylvatica* | 52.28551 | 5.9161 | 23 | 21/02/2008 | 31/12/2018 | EMS Brno DRL26 |
| Nabuurs.Lerink_Nieuw.Milligen_1559640587 | 4 | *Pinus sylvestris* | 52.21813 | 5.80761 | 44 | 21/02/2008 | 31/12/2018 | EMS Brno DRL26 |
| Nabuurs.Lerink_Quin_1559574338 | 3 | *Pinus sylvestris* | 51.38974 | 6.01242 | 11 | 12/05/2015 | 31/12/2018 | EMS Brno DRL26 |
| Nabuurs.Lerink_Pijpebrandje_1559571568 | 4 | *Pseudotsuga menziesii* | 52.25479 | 5.69874 | 20 | 01/03/2008 | 31/12/2018 | EMS Brno DRL26 |
| Nabuurs.Lerink_Schoonloerveld_1561129080 | 3 | *Pseudotsuga menziesii* | 52.88864 | 6.68476 | 22 | 01/03/2008 | 31/12/2018 | EMS Brno DRL26 |
| Nabuurs.Lerink_Starnumansbos_1561128295 | 4 | *Quercus robur* | 52.87508 | 5.55575 | 0 | 21/02/2008 | 31/12/2018 | EMS Brno DRL26 |
| Nabuurs.Lerink_Vijlnerbos_1559633858 | 5 | *Fagus sylvatica* | 50.46004 | 5.5802 | 213 | 21/02/2008 | 01/06/2019 | EMS Brno DRL26 |
| Nabuurs.Lerink_Zeesserveld_1559641099 | 2 | *Pinus sylvestris* | 52.50637 | 6.45437 | 5 | 21/02/2008 | 31/05/2019 | EMS Brno DRL26 |
| Oberhuber_Tschirgant.xeric_1559054911 | 3 | *Pinus sylvestris* | 47.2311 | 10.8444 | 740 | 11/03/2015 | 08/11/2018 | UMS DMS D6 |
| Oberhuber_Tschirgant_1558970324 | 16 | *Picea abies, Pinus sylvestris, Larix decidua* | 47.2314 | 10.8475 | 750 | 03/09/2010 | 08/11/2018 | Ecomatik DC2 |
| Obojes_Matsch_SF1_1559397841 | 4 | *Larix decidua, Pinus nigra* | 46.6777 | 10.5781 | 1160 | 22/03/2012 | 12/04/2019 | EMS Brno DRL26 |
| Obojes_Matsch_SF2_1559385166 | 4 | *Larix decidua* | 46.6944 | 10.6129 | 1715 | 22/03/2012 | 12/04/2019 | EMS Brno DRL26 |
| Obojes_Matsch_SF3_1559395615 | 4 | *Larix decidua* | 46.6977 | 10.6072 | 1990 | 23/05/2012 | 12/04/2019 | EMS Brno DRL26 |
| Obojes_Matsch_SF4_1559396501 | 6 | *Larix decidua, Pinus cembra* | 46.66987 | 10.6422 | 2030 | 23/05/2012 | 12/04/2019 | EMS Brno DR26 |
| Obojes_Matsch_SF5_1559398670 | 8 | *Larix decidua, Pinus cembra* | 46.7389 | 10.6884 | 2100 | 23/05/2012 | 12/04/2019 | EMS Brno DRL26 |
| Obojes_Matsch_WgN_1559400256 | 2 | *Pinus cembra* | 46.6961 | 10.6476 | 2320 | 01/07/2015 | 12/04/2019 | EMS Brno DRL26 |
| Obojes_Matsch_WgS_1559399655 | 4 | *Larix decidua* | 46.7389 | 10.6884 | 2230 | 26/06/2015 | 12/04/2019 | EMS Brno DRL26 |
| RATGEBER.BULTEAU.PEYRIE_Champenoux_1559248194 | 5 | *Quercus petraea* | 48.7208 | 6.34 | 240 | 01/01/2017 | 01/01/2019 | EMS Brno DRL26 |
| RATHGEBER.BULTEAU.PEYRIE_Ban.D.Harol_1559249461 | 4 | *Fagus sylvatica* | 48.104 | 6.2098 | 420 | 01/01/2017 | 01/01/2019 | EMS Brno DRL26 |
| Stangler.Kahle.Spiecker_Guenterstal_1559320776 | 13 | *Fagus sylvatica, Picea abies* | 47.95724 | 7.868327 | 750 | 01/01/2014 | 31/12/2018 | Trans-Tek Inc |
| Stangler.Kahle.Spiecker_Heibrain_1559320063 | 13 | *Fagus sylvatica, Picea abies* | 47.92656 | 7.87262 | 750 | 01/01/2014 | 31/12/2018 | Trans-Tek Inc |
| Stangler.Kahle.Spiecker_Schauinsland_1559319167 | 9 | *Fagus sylvatica, Picea abies* | 47.91342 | 7.903969 | 1250 | 01/01/2014 | 31/12/2018 | Trans-Tek Inc |
| Steppe.Luis.Salomon.Moreno.von.der.Crone_Experimental.forest.Aelmoeseneie | 3 | *Fagus sylvatica* | 50.975 | 3.8043 | 43.1 | 23/04/2014 | 30/12/2018 | Trans-Tek Inc |
| Stojanovic.Krejza_Lanzhot_1558681315 | 7 | *Fraxinus angustifolia, Quercus robur* | 48.6817 | 16.9464 | 155 | 24/03/2016 | 28/11/2018 | EMS Brno DRL26 |
| Svoboda_RO_BEL_002_2_1559085017 | 3 | *Abies alba, Fagus sylvatica* | 45.6369 | 24.9659 | 1241.37 | 23/09/2014 | 19/10/2018 | EMS Brno DRL26 |
| Svoboda_RO_BEL_004_1_1559057265 | 2 | *Abies alba, Fagus sylvatica* | 45.6414 | 24.965 | 1243.76 | 20/09/2014 | 19/10/2018 | EMS Brno DRL26 |
| Svoboda_RO_FA9_457_1559057830 | 3 | *Picea abies* | 45.5715 | 24.6046 | 1600.14 | 10/09/2013 | 19/10/2018 | EMS Brno DRL26 |
| Svoboda_RO_FA9_492_1559058338 | 2 | *Picea abies* | 45.5715 | 24.6067 | 1480.56 | 28/12/2013 | 19/10/2018 | EMS Brno DRL26 |
| Tobin.Osborne.Saunders.Zou_Dooary.Forest | 4 | *Picea sitchensis* | 52.9486 | -7.2644 | 249 | 01/01/2015 | 05/04/2019 | Natkon ZN11-T-WP |
| Trotsiuk_SK_JAV_016_1559059893 | 2 | *Picea abies* | 49.2132 | 20.1613 | 1476.11 | 23/03/2014 | 02/06/2018 | EMS Brno DRL26 |
| Trotsiuk_SK_JAV_062_1559061076 | 1 | *Picea abies* | 49.2166 | 20.1645 | 1417.13 | 19/10/2013 | 02/06/2018 | EMS Brno DRL26 |
| Uradnicek.Plichta_Pohansko_01_1558968267 | 14 | *Quercus robur* | 48.7216 | 16.90594 | 154 | 19/04/2018 | 16/01/2019 | TOMST |
| Urban_Bilovice_1557292040 | 5 | *Quercus petraea* | 49.2485 | 16.6867 | 320.61 | 06/07/2016 | 05/04/2019 | EMS Brno DRL26 |
| Urban_Utechov_1557287734 | 3 | *Fagus sylvatica* | 49.2798 | 16.6487 | 386.81 | 16/02/2017 | 05/04/2019 | EMS Brno DRL26 |
| Vejpustková_Benesovice_1559141173 | 6 | *Pinus sylvestris* | 49.7419 | 12.8614 | 385 | 01/01/2010 | 31/12/2018 | EMS Brno DR 26 |
| Vejpustková_Lazy_1559142795 | 6 | *Picea abies* | 50.0433 | 12.625 | 875 | 01/01/2010 | 31/12/2018 | EMS Brno DR 26 |
| Vejpustková_Vsetec_1559201018 | 6 | *Fagus sylvatica, Pinus sylvestris* | 49.23 | 14.3 | 615 | 01/01/2010 | 31/12/2018 | EMS Brno DR 26 |
| Vejpustková_Zelivka_1559202253 | 6 | *Picea abies* | 49.6753 | 15.2297 | 440 | 01/01/2010 | 31/12/2018 | EMS Brno DR 26 |
| Walthert_Alvaneu_1559299797 | 3 | *Pinus sylvestris* | 46.683 | 9.6411 | 1381 | 21/05/2014 | 31/12/2018 | Natkon ZN11-T-WP |
| Walthert_Neunkirch_1559300768 | 3 | *Fagus sylvatica* | 47.6837 | 8.5339 | 586.3 | 04/04/2014 | 27/12/2018 | Natkon ZN11-T-WP |
| Walthert_Saillon.Buche_1559303291 | 4 | *Fagus sylvatica* | 46.1713 | 7.1655 | 888.8 | 22/05/2015 | 31/12/2018 | Natkon ZN11-T-WP |
| Walthert_Saillon.Eiche_1559302615 | 3 | *Quercus pubescens* | 46.1701 | 7.1664 | 793.2 | 13/03/2014 | 31/12/2018 | Natkon ZN11-T-WP |
| Wilmking.Smiljanic.Scharnweber_Eldena_1563441190 | 18 | *Fagus sylvatica, Carpinus betulus, Quercus robur, Acer pseudoplatanus* | 54.0784 | 13.47553 | 7 | 01/04/2013 | 31/12/2018 | Ecomatik DR |
| Wilmking.Smiljanic.Scharnweber_Vilm_1563433143 | 13 | *Fagus sylvatica, Quercus robur, Acer pseudoplatanus* | 54.32637 | 13.54035 | 25 | 01/12/2014 | 31/12/2018 | Ecomatik DR |
| Zin.Kuberski.Sterenczak_BF_03_1560196768 | 5 | *Pinus sylvestris* | 52.69457 | 23.82799 | 177.89 | 23/04/2015 | 31/12/2018 | EMS Brno DRL26 |
| Zin.Kuberski.Sterenczak_BF_05_1560198208 | 2 | *Alnus glutinosa* | 52.69457 | 23.82799 | 165.86 | 24/04/2015 | 31/12/2018 | EMS Brno DRL26 |
| Zin.Kuberski.Sterenczak_BF_09_1560198723 | 6 | *Betula L., Tilia cordata* | 52.69457 | 23.82799 | 178.75 | 30/04/2015 | 31/12/2018 | EMS Brno DRL26 |
| Zin.Kuberski.Sterenczak_BF_10_1560199317 | 6 | *Fraxinus excelsior, Acer platanoides* | 52.69457 | 23.82799 | 171.8 | 15/05/2015 | 31/12/2018 | EMS Brno DRL26 |
| Zin.Kuberski.Sterenczak_BF_12_1560370506 | 1 | *Tilia cordata* | 52.69457 | 23.82799 | 165 | 01/05/2015 | 31/12/2018 | EMS Brno DRL26 |
| Zin.Kuberski.Sterenczak_BF_15_1560370880 | 3 | *Quercus robur* | 52.69457 | 23.82799 | 164.27 | 03/06/2015 | 31/12/2018 | EMS Brno DRL26 |
| Zin.Kuberski.Sterenczak_BF_17_1560371380 | 5 | *Betula L., Pinus sylvestris* | 52.69457 | 23.82799 | 161.89 | 17/05/2015 | 31/12/2018 | EMS Brno DRL26 |
| Zin.Kuberski.Sterenczak_BF_19_1560371753 | 3 | *Fraxinus excelsior* | 52.69457 | 23.82799 | 166.88 | 24/05/2015 | 31/12/2018 | EMS Brno DRL26 |
| Zin.Kuberski.Sterenczak_BF_20_1560372273 | 4 | *Alnus glutinosa* | 52.69457 | 23.82799 | 160.02 | 20/05/2015 | 31/12/2018 | EMS Brno DRL26 |
| Zin.Kuberski.Sterenczak_BF_21_1560372607 | 4 | *Acer platanoides* | 52.69457 | 23.82799 | 175.44 | 21/05/2015 | 31/12/2018 | EMS Brno DRL26 |
| Zin.Kuberski.Sterenczak_BF_24_1560372991 | 3 | *Ulmus L.* | 52.69457 | 23.82799 | 170 | 28/05/2015 | 31/12/2018 | EMS Brno DRL26 |
| Zin.Kuberski.Sterenczak_BF_27_1560373938 | 3 | *Pinus sylvestris* | 52.69457 | 23.82799 | 159.22 | 08/05/2015 | 31/12/2018 | EMS Brno DRL26 |
| Zin.Kuberski.Sterenczak_BF_33_1560374435 | 5 | *Picea abies* | 52.69457 | 23.82799 | 164.67 | 30/04/2015 | 31/12/2018 | EMS Brno DRL26 |
| Zweifel_Davos.Seehornwald_1562742428 | 9 | *Picea abies* | 46.81522 | 9.8558 | 1650 | 01/01/2013 | 31/12/2018 | Natkon ZN11-T-WP |

Online Resource Table S2. The tree species wood anatomy based on literature.

| **Species** | **Wood anatomy type** | **References** |
| --- | --- | --- |
|  | (diffuse-porous/ring-porous/coniferous) |  |
| *Abies alba* | Coniferous | Schoch et al. 2004 |
| *Acer platanoides* | Diffuse-porous | Schoch et al. 2004 |
| *Acer psuedoplatanus* | Diffuse-porous | Schoch et al. 2004 |
| *Ailanthus altissima* | Ring-porous | Huber et al. 2025 |
| *Alnus glutinosa* | Diffuse-porous | Schoch et al. 2004 |
| *Betula sp.* | Diffuse-porous | Schoch et al. 2004 |
| *Carpinus betulus* | Diffuse-porous | Schoch et al. 2004 |
| *Castanea sativa* | Ring-porous | Schoch et al. 2004 |
| *Corylus colurna* | Diffuse-porous | Zeidler 2012 |
| *Fagus sylvatica* | Diffuse-porous | Schoch et al. 2004 |
| *Fraxinus excelsior* | Ring-porous | Schoch et al. 2004 |
| *Fraxinus angustifolia* | Ring-porous | Noyer et al. 2023 |
| *Juglans nigra* | Diffuse-porous | Peters, McFadden and Montgomery, 2010 |
| *Juniperus thurifera* | Coniferous | Gastón González et al., 2016 |
| *Larix decidua* | Coniferous | Schoch et al. 2004 |
| *Metasequoia glyptostroboides* | Coniferous | Williams 2005 |
| *Picea abies* | Coniferous | Schoch et al. 2004 |
| *Picea sitchensis* | Coniferous | Harris 1984 |
| *Pinus cembra* | Coniferous | Schoch et al. 2004 |
| *Pinus nigra* | Coniferous | Schoch et al. 2004 |
| *Pinus sylvestris* | Coniferous | Schoch et al. 2004 |
| *Pseudotsuga menziesii* | Coniferous | Schoch et al. 2004 |
| *Quercus faginea* | Ring-porous | Sousa et al., 2014 |
| *Quercus ilex* | Diffuse-porous | Denk et al. 2017 |
| *Quercus petraea* | Ring-porous | Schoch et al. 2004 |
| *Quercus pubescens* | Ring-porous | Schoch et al. 2004 |
| *Quercus robur* | Ring-porous | Schoch et al. 2004 |
| *Tilia cordata* | Diffuse-porous | Schoch et al. 2004 |
| *Ulmus sp.* | Ring-porous | Schoch et al. 2004 |

References to the Online Resource Table S2.

Denk T, Grimm GW, Manos PS, et al (2017) An Updated Infrageneric Classification of the Oaks: Review of Previous Taxonomic Schemes and Synthesis of Evolutionary Patterns. In: Gil-Pelegrín E, Peguero-Pina JJ, Sancho-Knapik D (eds) Oaks Physiological Ecology. Exploring the Functional Diversity of Genus Quercus L. Springer International Publishing, Cham, pp 13–38. Available at: https://doi.org/10.1007/978-3-319-69099-5_2

Gastón Gonzáles, A, García-Viñas, JI., Saura, S, et al (2016) Juniperus thurifera in Europe: distribution, habitat, usage and threats, in: European Commission. Joint Research Centre. (2016) European atlas of forest tree species. LU: Publications Office. Available at: https://data.europa.eu/doi/10.2760/776635 (Accessed: 7 October 2025).

Harris, AS (1984) Sitka spruce (Picea sitchensis (Bong.) Carr.). FS-265. Available at: https://research.fs.usda.gov/treesearch/32205

Huber, C, Teischinger, A, Stingl, R, Pramreiter, M, et al (2025) Wood Technological Properties of the Tree of Heaven (Ailanthus altissima (Mill.) Swingle) and Studies on Possible Applications. Forest Products Journal 75(2):109–119. Available at: https://doi.org/10.13073/FPJ-D-24-00050

Noyer E, Stojanović M, Horáček P, Pérez‐de‐Lis G (2023) Toward a better understanding of angiosperm xylogenesis: a new method for a cellular approach. New Phytol 239:792–805. <https://doi.org/10.1111/nph.18959>

Peters EB, McFadden JP, Montgomery RA (2010) Biological and environmental controls on tree transpiration in a suburban landscape. J Geophys Res Biogeosciences 115:1–13. https://doi.org/10.1029/2009JG001266

Schoch, W, Heller, I, Schweingruber, FH, et al (2004) Wood anatomy of central European Species. Online version: [www.woodanatomy.ch](http://www.woodanatomy.ch) (Accessed: 8 December 2025)

Sousa VB, Cardoso S, Pereira H (2014) Age trends in the wood anatomy of Quercus faginea. IAWA J 35:293–306. https://doi.org/10.1163/22941932-00000067

Williams CJ (2005) Ecological Characteristics of Metasequoia glyptostroboides. In: LePage BA, Williams CJ, Yang H (eds) The Geobiology and Ecology of Metasequoia. Springer-Verlag, Berlin/Heidelberg, pp 285–304

Zeidler A (2012) Variation of wood density in Turkish hazel (Corylus colurna L.) grown in the Czech Republic. J For Sci 58:145–151. https://doi.org/10.17221/73/2011-JFS

Online Resource Table S3. Comparison of the performance of different TWD characteristics for model building. Exceeding TWD length refers to the length of TWD period that exceeds 120 h. All models were Poisson distributed.

| **TWD Characteristics** | **Model function** | **AIC values** |
| --- | --- | --- |
| Lowest TWD_min_ | Exceeding TWD period length ~ lowest TWD_min_ + (1\| site/tree) + (1\|year) | 59082.49 |
| Mean TWD_min_ | Exceeding TWD period length ~ mean TWD_min_ + (1\| site/tree) + (1\|year) | 53270.80 |
| Highest TWD_min_ | Exceeding TWD period length ~ highest TWD_min_ + (1\| site/tree) + (1\|year) | 49876.03 |
| Lowest TWD_avg_ | Exceeding TWD period length ~ lowest TWD_avg_ + (1\| site/tree) + (1\|year) | 59122.38 |
| Mean TWD_avg_ | Exceeding TWD period length ~ mean TWD_avg_ + (1\| site/tree) + (1\|year) | 53793.94 |
| Highest TWD_avg_ | Exceeding TWD period length ~ highest TWD_avg_ + (1\| site/tree) + (1\|year) | 50244.07 |
| Lowest TWD_max_ | Exceeding TWD period length ~ lowest TWD_max_ + (1\| site/tree) + (1\|year) | 59090.38 |
| Mean TWD_max_ | Exceeding TWD period length ~ mean TWD_max_ + (1\| site/tree) + (1\|year) | 54696.20 |
| Highest TWD_max_ | Exceeding TWD period length ~ highest TWD_max_ + (1\| site/tree) + (1\|year) | 51580.59 |

Online Resource Table S4. Comparison the model performance with different climatic variables used. Exceeding TWD length refers to the length of TWD period that exceeds 120 h. All models had negative binomial distribution.

| **Climate variables used** | **Model function** | **AIC values** |
| --- | --- | --- |
| Temperature | Exceeding TWD period length ~ highest TWDmin * wood type + highest temperature * wood type + recurrence of TWD period * wood type + (1\| site/tree) + (1\|year) | 37117.55 |
| Temperature and SPEI | Exceeding TWD period length ~ highest TWDmin * wood type + highest temperature * wood type + highest temperature * lowest SPEI + recurrence of TWD period * wood type + (1\| site/tree) + (1\|year) | 36997.35 |
| Maximum VPD | Exceeding TWD period length ~ highest TWDmin * wood type + maximum VPD * wood type + recurrence of TWD period * wood type + (1\| site/tree) + (1\|year) | 37501.43 |
| Maximum VPD and temperature | Exceeding TWD period length ~ highest TWDmin * wood type + maximum VPD * wood type + highest temperature * wood type + recurrence of TWD period * wood type + (1\| site/tree) + (1\|year) | 37122.49 |

Online Resource Table S5. Comparison of the models with different distributions, their AIC values and dispersion test.

| **Model distribution** | **Model function** | **AIC values** | **Dispersion test** |
| --- | --- | --- | --- |
| Poisson | Exceeding TWD period length ~ highest TWDmin * wood type + highest temperature * wood type + highest temperature * lowest SPEI + recurrence of TWD period * wood type + (1\| site/tree) + (1\|year) | 45431.23 | Dispersion 1.4132, p-value = 0.192 |
| Poisson with random ID effect | Exceeding TWD period length ~ highest TWDmin * wood type + highest temperature * wood type + highest temperature * lowest SPEI + recurrence of TWD period * wood type + (1\| site/tree) + (1\|year) + (1\|index) | 37088.46 | Dispersion 0.54015, p-value < 0.01 |
| Zero-inflated Poisson | Exceeding TWD period length ~ highest TWDmin * wood type + highest temperature * wood type + highest temperature * lowest SPEI + recurrence of TWD period * wood type + (1\| site/tree) + (1\|year),  zi ~ lowest SPEI + highest temperature + highest TWDmin + wood type + recurrence of TWD period) | 44485.04 | Dispersion 1.4005, p-value = 0.16 |
| Negative binomial | Exceeding TWD period length ~ highest TWDmin * wood type + highest temperature * wood type + highest temperature * lowest SPEI + recurrence of TWD period * wood type + (1\| site/tree) + (1\|year) | 36997.35 | Dispersion 0.77319. p-value = 0.288 |

Online Resource Table S6. The model configurations and dataset used in the sensitivity analyses. The aspects differing from the model used for analysis are shown in bold.

| Sensitivity analysis | Model configuration |
| --- | --- |
| Testing the effect of including the dendrometer model in the fixed-effects | Exceeding TWD period length ~ highest TWDmin * wood type + highest temperature * wood type + highest temperature * lowest SPEI + recurrence of TWD period * wood type + **dendrometer model** + (1\| site/tree) + (1\|year)  Data used: full data set |
| Testing the effect of excluding sites below the 45° latitude | Exceeding TWD period length ~ highest TWDmin * wood type + highest temperature * wood type + highest temperature * lowest SPEI + recurrence of TWD period * wood type + (1\| site/tree) + (1\|year)  **Data used: subset of the data set without sites below 45° latitude** |
| Testing the effect of different TWD period definitions | Exceeding TWD period length ~ highest TWDmin * wood type + highest temperature * wood type + highest temperature * lowest SPEI + recurrence of TWD period * wood type + (1\| site/tree) + (1\|year)  **Data used: Subsets of the data with the different TWD periods including only the trees present in each of the subsets (subset TWD period defined as at least 72, 120, and 168 hours)** |
| Testing the effect of including the timing of the TWD period in fixed-effects | Exceeding TWD period length ~ highest TWDmin * wood type + highest temperature * wood type + highest temperature * lowest SPEI + recurrence of TWD period * wood type + **starting month of the TWD period * wood type** + (1\| site/tree) + (1\|year)  Data used: full data set |

Online Resource Table S7. Results of the Generalized Linear Mixed Effects Model (GLMM; negative binomial error distribution with log link) explaining the length of a TWD period. The fixed-effects structure was: Exceeding TWD period length ~ highest TWDmin * wood type + highest temperature * wood type + highest temperature * lowest SPEI + recurrence of TWD period * wood type, with random intercepts for site/tree and year. (1| site/tree) + (1|year). Estimates are presented on the log scale (raw estimates) and as exponentiated coefficients (incidence rate ratios, IRRs). A TWD period was defined as a continuous period of at least 120 h during which TWD remained above zero. To model variation beyond this minimum duration, four days were subtracted from each TWD period before analysis. Expected values were therefore back-transformed by adding four days to model predictions, yielding total TWD period lengths in days.

| Variable | Estimate (log scale) | SE | p-values | IRR (= exp(β)) | Expected value, exp(β_0_ + β) | Expected duration (days)* |
| --- | --- | --- | --- | --- | --- | --- |
| Intercept | 1.242 | 0.0614 | < 0.001 | 3.464 | 3.464 | 7.464 |
| Highest TWD_min_ | 0.531 | 0.0203 | < 0.001 | 1.701 | 5.892 | 9.892 |
| Highest temperature | 0.366 | 0.0176 | < 0.001 | 1.441 | 4.992 | 8.992 |
| Lowest SPEI | -0.030 | 0.0180 | 0.101 | 0.971 | 3.364 | 7.364 |
| Wood type: Diffuse | -0.404 | 0.0988 | < 0.001 | 0.668 | 2.314 | 6.314 |
| Wood type: Ring | -0.170 | 0.0848 | < 0.050 | 0.843 | 2.920 | 6.920 |
| Recurrence of TWD period | 0.013 | 0.0097 | 0.197 | 1.013 | 3.509 | 7.509 |
| Highest temperature * lowest SPEI | -0.082 | 0.0127 | < 0.001 | 0.921 | 3.190 | 7.190 |
| Highest TWD_min_ * Wood type: Diffuse | -0.181 | 0.0484 | < 0.001 | 0.835 | 2.892 | 6.892 |
| Highest TWD_min_ * Wood type: Ring | 0.002 | 0.0379 | 0.957 | 1.002 | 3.471 | 7.471 |
| Wood type: Diffuse * highest temperature | 0.009 | 0.0434 | 0.843 | 1.009 | 3.495 | 7.495 |
| Wood type: Ring * highest temperature | 0.009 | 0.0321 | 0.784 | 1.009 | 3.495 | 7.495 |
| Wood type: Diffuse * recurrence of TWD period | 0.113 | 0.0327 | < 0.001 | 1.120 | 3.880 | 7.880 |
| Wood type: Ring * recurrence of TWD period | 0.011 | 0.0210 | 0.586 | 1.012 | 3.506 | 7.506 |

* Calculated as exp(β₀ + β) + 4, where four days were subtracted from TWD period lengths before model fitting.
**Note:** Because TWD periods can begin and end at any hour of the day, the first and last calendar days may represent only partial days of water deficit. Consequently, a TWD period meeting the minimum duration criterion of 120 h may span more calendar dates than the equivalent number of complete days.

Online Resource Table S8. The model coefficients of the TWD definition sensitivity analysis with a TWD period remaining above 0 for at least 72 hours and a TWD period remaining above 0 for at least 168 hours.

|  | TWD definition: TWD above zero for at least 72 hours | | | TWD definition: TWD above zero for at least 168 hours | | |
| --- | --- | --- | --- | --- | --- | --- |
| Variable | Estimate | Standard error | p-values | Estimate | Standard error | p-values |
| Intercept | 1.325 | 0.0568 | < 0.001 | 1.153 | 0.0690 | < 0.001 |
| Highest TWD_min_ | 0.674 | 0.0179 | < 0.001 | 0.476 | 0.0238 | < 0.001 |
| Highest temperature | 0.376 | 0.0127 | < 0.001 | 0.396 | 0.0238 | < 0.001 |
| Lowest SPEI | -0.041 | 0.0117 | < 0.001 | -0.097 | 0.0247 | < 0.001 |
| Wood type: Diffuse | -0.517 | 0.0758 | < 0.001 | -0.500 | 0.1259 | < 0.001 |
| Wood type: Ring | -0.114 | 0.0743 | 0.127 | -0.076 | 0.0998 | 0.445 |
| Recurrence of TWD period | 0.014 | 0.0053 | < 0.050 | 0.005 | 0.0158 | 0.754 |
| Highest temperature * lowest SPEI | -0.044 | 0.0087 | < 0.001 | -0.063 | 0.0169 | < 0.001 |
| Highest TWD_min_ * Wood type: Diffuse | -0.137 | 0.0432 | < 0.050 | -0.139 | 0.0569 | < 0.050 |
| Highest TWD_min_ * Wood type: Ring | -0.039 | 0.0326 | 0.229 | -0.040 | 0.0446 | 0.371 |
| Wood type: Diffuse * highest temperature | 0.033 | 0.0299 | 0.273 | -0.063 | 0.0569 | 0.270 |
| Wood type: Ring * highest temperature | 0.005 | 0.0236 | 0.834 | -0.024 | 0.0418 | 0.559 |
| Wood type: Diffuse * recurrence of TWD period | 0.083 | 0.0150 | < 0.001 | 0.257 | 0.0553 | < 0.001 |
| Wood type: Ring * recurrence of TWD period | -0.004 | 0.0118 | 0.716 | 0.031 | 0.0338 | 0.354 |

Online Resource Figure S1. Distribution of mean elevation-corrected temperature (see method) during TWD periods across years, wood types, and the total number of TWD periods per year. Each panel represents one annual frequency of TWD periods (1 to 8 periods per year), while rows correspond to coniferous, diffuse-porous, and ring-porous tree species. For each year, colored bars show the observed range (minimum to maximum) of mean elevation-corrected temperature (°C). The black filled circles indicate the mean, and the white circles indicate the median. Numbers above each year denote the number of individual trees contributing observations for that year and panel. The figure illustrates the distribution and temporal coverage of the 6,882 TWD periods analyzed across 693 trees from 130 sites between 2003 and 2018.

Online Resource Figure S2. Distribution of the maximum daily temperature reached during each TWD period. The histogram shows the maximum daily temperature (°C) during each of the 6882 TWD periods analyzed. Dashed vertical lines indicate heat thresholds of 30°C, 35°C, and 40°C. Most TWD periods (69.8%) experienced maximum temperatures below 30°C, whereas 25.7% reached 30–35°C, 4.5% reached 35–40°C, and only one single TWD period exceeded 40°C. This indicates that the observed relationship between temperature and TWD duration is primarily driven by variation within the typical European summer temperature range rather than by rare extreme heat events.

Online Resource Figure S3. Distribution of mean SPEI during TWD periods across years, wood type, and the total number of TWD periods per year. Colored bars represent the observed range, black filled circles the mean, white circles the median, and numbers above each year the number of individual trees contributing observations as in Online Resource Figure S1.

Online Resource Figure S4. Distribution of mean site-standardized temperature during TWD periods across years, wood type, and the total number of TWD periods per year. Temperatures were standardized separately for each site by subtracting the site mean and dividing by the site standard deviation, allowing comparison of relative temperature anomalies among sites spanning broad climatic gradients. This standardization enables comparison of relative warm anomalies across the European climatic gradient. The corresponding analyses using absolute temperatures (°C) yielded qualitatively similar results and are presented in Online Resource Figure S1. Colored bars represent the observed range, black filled circles the mean, white circles the median, and numbers above each year the number of individual trees contributing observations as in Online Resource Figure 1.

Online Resource Figure S5. Distribution of TWD period lengths across years, wood types, and the total number of TWD periods per year. Each panel represents one annual frequency of TWD periods (1 to 8 periods per year), while rows correspond to coniferous, diffuse-porous, and ring-porous tree species. For each year, colored bars show the observed range (minimum to maximum) of TWD period lengths (days). Colored bars represent the observed range, black filled circles the mean, white circles the median, and numbers above each year the number of individual trees contributing observations as in Online Resource Figure S1.

Online Resource Figure S6. The relationship between a TWD variable and the exceeding TWD period length based on the Online Resource Table S3. In each panel, the x-axis shows the length of the TWD period that exceeds the definition of a TWD period, i.e., 5 days. The y-axis shows a) the highest TWD_max_, b) the mean TWD_max_, c) the lowest TWD_max_, d) the highest TWD_avg_, e) the mean TWD_avg_, f) the lowest TWD_avg_, g) the highest TWD_min_, h) the mean TWD_min_, and i) the lowest TWD_min_. Note that the scales of y-axes are different. In each panel, the AIC values from the Online Resource Table S3 and the pairwise Spearman correlation is shown.

Online Resource Figure S7. The pairwise Spearman correlation heatmap of the different TWD variables and the exceeding TWD period length.

Online Resource Figure S8. The performance of the residuals of the selected model, created with DHARMa package. Kolmogorov-Smirnov (KS test) test is sensitive to large sample sizes. We performed an additional Cramér–von Mises test that was non-significant, indicating that model residuals are consistent with uniformity.

Online Resource Figure S9. The effect of the interaction between maximum temperature and SPEI and the recurrence of the TWD period on the length of the TWD period for different wood types.

Online Resource Figure S10. The difference in model coefficients with the different definitions for a TWD period: 3d = TWD remains above zero for at least 72 hours, 5d = TWD remains above zero for at least 120 hours, and 7d = TWD remains above zero for at least 168 hours. The y-axis shows the model coefficient estimates for the different model terms.
